# Integrative Metabolomics Reveals Deep Tissue and Systemic Metabolic Remodeling in Glioblastoma

**DOI:** 10.3390/cancers13205157

**Published:** 2021-10-14

**Authors:** Vianney Gilard, Justine Ferey, Florent Marguet, Maxime Fontanilles, Franklin Ducatez, Carine Pilon, Céline Lesueur, Tony Pereira, Carole Basset, Isabelle Schmitz-Afonso, Frédéric Di Fioré, Annie Laquerrière, Carlos Afonso, Stéphane Derrey, Stéphane Marret, Soumeya Bekri, Abdellah Tebani

**Affiliations:** 1 Department of Neurosurgery, UNIROUEN, CHU Rouen, INSERM U1245, Normandie University, 76000 Rouen, France; Vianney.gilard@chu-rouen.fr; 2 Department of Metabolic Biochemistry, UNIROUEN, CHU Rouen, INSERM U1245, Normandie University, 76000 Rouen, France; justine.ferey@inrae.fr (J.F.); franklin.ducatez@gmail.com (F.D.); carine.pilon@chu-rouen.fr (C.P.); celine.lesueur@chu-rouen.fr (C.L.); abdellah.tebani@chu-rouen.fr (A.T.); 3 Department of Pathology, UNIROUEN, CHU Rouen, INSERM U1245, Normandie University, 76000 Rouen, France; florent.marguet@chu-rouen.fr (F.M.); carole.basset@chu-rouen.fr (C.B.); annie.laquerriere@chu-rouen.fr (A.L.); 4 Institut de Biologie Clinique, CHU Rouen, 76000 Rouen, France; maxime.fontanilles@chb.unicancer.fr (M.F.); tony.pereira@chu-rouen.fr (T.P.); 5 INSA Rouen, CNRS IRCOF, 1 Rue TesnieÌre, COBRA UMR 6014 Et FR 3038 University Rouen, Normandie University, CEDEX, 76821 Mont-Saint-Aignan, France; isabelle.schmitz-afonso@univ-rouen.fr (I.S.-A.); carlos.afonso@univ-rouen.fr (C.A.); 6 Intensive Care and Neuropediatrics, Department of Neonatal Pediatrics, INSERM U1245, CHU Rouen, UNIROUEN, Normandie University, 76000 Rouen, France; stephane.marret@chu-rouen.fr; 7 Normandy Centre for Genomic and Personalized Medicine, IRON Group, INSERM U1245, UNIROUEN, Normandie University, 76000 Rouen, France; Frederic.difiore@chu-rouen.fr; 8 Department of Medical Oncology, Cancer Centre Henri Becquerel, Rue d’Amiens, 76000 Rouen, France; 9 Department of Neurosurgery, CHU Rouen, INSERM U1073, UNIROUEN, Normandie University, 76000 Rouen, France; stephane.derrey@chu-rouen.fr

**Keywords:** glioblastoma, high-grade glioma, brain tumor, neuro-oncology, metabolomics, omics, mass spectrometry, machine learning

## Abstract

**Simple Summary:**

This study aims to explore metabolic remodeling in plasma and tissue samples in patients with glioblastoma through an integrated targeted and untargeted metabolomics-based strategy. We report phospholipids, sphingomyelins, acylcarnitines, and acylglycerols as key impaired metabolic classes in glioblastoma.

**Abstract:**

(1) Background: Glioblastoma is the most common malignant brain tumor in adults. Its etiology remains unknown in most cases. Glioblastoma pathogenesis consists of a progressive infiltration of the white matter by tumoral cells leading to progressive neurological deficit, epilepsy, and/or intracranial hypertension. The mean survival is between 15 to 17 months. Given this aggressive prognosis, there is an urgent need for a better understanding of the underlying mechanisms of glioblastoma to unveil new diagnostic strategies and therapeutic targets through a deeper understanding of its biology. (2) Methods: To systematically address this issue, we performed targeted and untargeted metabolomics-based investigations on both tissue and plasma samples from patients with glioblastoma. (3) Results: This study revealed 176 differentially expressed lipids and metabolites, 148 in plasma and 28 in tissue samples. Main biochemical classes include phospholipids, acylcarnitines, sphingomyelins, and triacylglycerols. Functional analyses revealed deep metabolic remodeling in glioblastoma lipids and energy substrates, which unveils the major role of lipids in tumor progression by modulating its own environment. (4) Conclusions: Overall, our study demonstrates in situ and systemic metabolic rewiring in glioblastoma that could shed light on its underlying biological plasticity and progression to inform diagnosis and/or therapeutic strategies.

## 1. Introduction

Glioblastoma (GBM) is the most common malignant brain tumor in adults [1]. Its incidence, 2 to 5 per 100,000 [1,2], is still increasing [3,4], and its etiology remains unknown in most cases. Glioblastoma pathogenesis consists of a progressive infiltration of the white matter by tumoral cells leading to progressive neurological deficit, epilepsy, and/or intracranial hypertension [5,6]. Multimodal magnetic resonance imaging (MRI) is the first-line investigation to diagnose glioblastoma and guides treatment strategies [7]. The current standard of care in the management of glioblastoma is based on surgical resection or biopsy followed by adjuvant chemotherapy and radiotherapy [8,9,10]. Despite many therapeutic trials [11,12,13], the prognosis of glioblastoma is poor, with a mean survival between 15 to 17 months [14]. Given this poor prognosis, there is an urgent need for a better understanding of the underlying mechanisms of glioblastoma to unveil new diagnosis strategies and therapeutic targets through a deeper understanding of its biology. During the last few years, Precision Medicine (PM) has been a new paradigm that has opened a new era in personalized diagnosis and therapeutic approaches in oncology [15,16,17,18]. It is a tailored approach in disease management driven by the patient’s attributes to deliver personalized healthcare. The concept of PM is penetrating steadily into the clinical setting with increasing evidence in different areas, including oncology [15], autoimmunity [19], or inborn errors of metabolism [20]. The PM surge is shaped by omics-based technologies and the data-driven medicine revolution [21,22,23]. Omics are technologies that allow for a multilayer molecular deciphering of the cell, tissue, and organism in a holistic fashion. These technologies have been explored in various avenues of medicine and health sciences, such as screening, diagnosis, and prognosis [24]. Given their capacity to interrogate biology at an unprecedented depth and breath, omics are also reshaping the biomarker discovery landscape [25]. Metabolomics, one of these technologies [26], directly emulates the biochemical activity of a biological system with high time sensitivity and spatial resolution. Metabolomics is the closest biological information layer to the phenotype, given its position in the downstream information flow. Thus, interrogating the metabolome, which is a set of metabolites in a given biological system, is an appealing opportunity to parse the biological process. Two main technologies are used in metabolomics: mass spectrometry (MS) and nucleic magnetic resonance (NMR) [27,28]. The latter is nondestructive and robust; however, the former exhibits higher sensitivity and broader metabolome coverage. High-resolution mass spectrometry (HRMS) coupled to other analytical modalities, such as liquid chromatography (LC-MS) or MALDI-MS, offers a great opportunity to achieve higher metabolic coverage, thus, biological scope. Furthermore, ultra-high resolution mass spectrometry instruments based on Fourier-transform ion cyclotron resonance (FTICR) exhibits unique resolution, sensitivity, and specificity [29,30,31]. Thus, this technology offers unprecedented exhaustive metabolome coverage and non-ambiguous molecular formula assignments [31]. Thus, disease-related metabolic phenotype could be described by retrieving metabolomic profiles through biospecimens, such as blood, urine, tissue samples, or cerebrospinal fluid [32,33,34]. Previous human-based studies have described glioblastoma-related metabolites [35,36,37,38,39,40,41,42,43], and to the best of our knowledge, no study has reported results based on both plasma and tissue analysis from the same glioblastoma patients. The aim of this study is to explore the glioblastoma biological landscape through an integrated metabolomics analysis of plasma and tissue samples of glioblastoma patients. This strategy presents a great opportunity to explore both the in-situ and systemic metabolic phenotypes and unveil predictive metabolomic patterns that could help early non-invasive glioblastoma diagnosis at presentation or recurrence. It might also provide new insights regarding glioblastoma biology and ultimately guide targeted treatment strategies.

## 2. Materials and Methods

### 2.1. Patients and Blood Samples

From January 2020 to November 2020, twenty-nine patients with histologically documented glioblastoma were selected for the study. All patients were referred to our neurosurgery department, and resection or biopsies of the tumor were performed in all cases. Plasma and tissue samples were collected at surgery before initiating any treatment. Medical charts were reviewed, and the following data were collected: gender, age at diagnosis, past medical history, revealing symptoms, radiological characteristics, type of surgery performed, and histological and biological characteristics of the tumor. All samples used in this study belong to a declared collection located in the Pathology Department (Prof. Annie Laquerrière) of Rouen University Hospital in accordance with the relevant guidelines and regulations and with the permission of the local authorities. All procedures performed in studies involving human participants were in accordance with the ethical standards of the institutional and/or national research committee and with the 1964 Helsinki Declaration and its later amendments or comparable ethical standards.

### 2.2. Targeted Metabolomics

#### 2.2.1. Reagents

All reagents, internal and calibration standards, quality controls, test mix, and a patented 96-well filter plate required for the AbsoluteIDQ^®^p180 analysis are included in the kit or provided by Biocrates Life Science AG (Innsbruck, Austria).

#### 2.2.2. Sample Preparation

Sample preparation was carried out according to the manufacturer’s protocol [44,45]. Briefly, 10 µL of plasma were transferred to the upper 96-well plate and dried under a nitrogen stream. Thereafter, 50 µL of a 5% PITC solution was added to derivatize amino acids and biogenic amines. After incubation, the spots were dried again before the metabolites were extracted using 5 mM ammonium acetate in methanol (300 µL) into the lower 96-well plate for analysis after further dilution using the MS running solvent A. Quantification was carried out using internal standards and a calibration curve [44,45]. The full list of 188 measured metabolites is presented in Table S1.

#### 2.2.3. Liquid Chromatography and Tandem Mass Spectrometry

The AbsoluteIDQ^®^ p180 kit is a fully automated assay based on phenyl isothiocyanate (PITC) derivatization of the target analytes in bodily fluids using internal standards for quantitation. Amino acids and biogenic amines are determined in LC-MS mode, acylcarnitines, phospholipids, sphingomyelins, and the sum of hexoses are analyzed in flow injection analysis (FIA). The analyses were performed following the instructions of the kit manufacturer using the liquid chromatography instrument prominence Shimadzu UFLC System (Shimadzu, Prominence, Kyoto, Japan) coupled to the 4000 Qtrap mass spectrometer (Sciex, Framingham, MA, USA) with an electrospray ion source. A system suitability test was conducted before each batch of the samples (analysis of a standard mixture) to warm up the LC-MS/MS system and check the inter-day performance of the system. Data acquisition and processing were performed using the Analyst 1.5 software (Sciex, Framingham, MA, USA). Twenty microliters of the sample extract were used in the flow injection analysis (FIA) in positive mode to capture acylcarnitines, glycerophospholipids, and sphingolipids, while hexoses were monitored in a subsequent negative mode run. All FIA injections were carried out using the Biocrates MS Running Solvent. All metabolites were identified and quantified using isotopically labeled internal standards and multiple reaction monitoring (MRM) as optimized and provided by Biocrates Life Sciences AG (Innsbruck, Austria).

### 2.3. Untargeted Metabolic Phenotyping

#### 2.3.1. Reagents

Water, acetonitrile, isopropanol, and formic acid were optima LCMS grade and purchased from Fisher Chemicals. Ammonium formate was LCMS grade (Sigma–Aldrich, Saint-Quentin Fallavier, France). Leucine Enkephalin (Sigma–Aldrich, Saint-Quentin Fallavier, France) at a concentration of 2 mg/mL (in isopropanol/water, 50/50) was used as a reference for mass measurements. Poly-DL-alanine was prepared in 50:50 (*v*/*v*) water/acetonitrile at 10 mg/L and used for ion mobility cell calibration.

#### 2.3.2. Sample Preparation

Human plasmas were prepared using a liquid biphasic approach. Lipids were extracted from 100 µL of plasma by adding 280 µL chloroform, 140 µL methanol, and 84 µL water. The mixtures were vortex-mixed, cooled in ice for 15 minutes, and centrifuged 10 min at 12,000 rpm. The bottom phase was removed, transferred to glass UPLC vials, and N_2_ evaporated. The resulting phase was re-suspended in 200 µL ACN/H_2_O/IPrOH (1:1:1). A pooled quality control (QC) was prepared by mixing 5 µL of each plasma sample. Four QC dilutions at 1:2, 1:4, 1:8, and 1:16 were prepared from the QC sample. Samples were stored at −20°C before use.

#### 2.3.3. Chromatographic Conditions

The liquid chromatography separation was performed with a UHPLC system (Dionex Ultimate 3000 UPLC+, Thermo Scientific, San Jose, CA, USA). Separation was carried out at 50 °C using a 1.0 × 100 mm Acquity UPLC HSS T3 column (Waters), with a particle size of 1.8 µm, equipped with a 0.2 µm prefilter. Mobile phase A consisted of ACN/H_2_O (40/60) containing 10 mM ammonium formate; mobile phase B consisted of IPrOH/ACN (90/10). Both solvents contained 0.1% formic acid. The flow rate was maintained at 100 µL min^−1^. A mobile phase gradient separation was performed over 20 min according to the following steps: 10% B at 0 min, 56% B at 2 min, 75% B at 10 min, 99% B at 12–15 min, 10% B at 16–20 min. Sample analysis order was randomized to avoid the potential for confounding critical variables with analytical run order effects.

#### 2.3.4. Ion Mobility and Mass Spectrometry

The U-HPLC system was coupled to a hybrid quadrupole orthogonal time-of-flight (TOF) mass spectrometer (SYNAPT G2 HDMS, Waters MS Technologies, Manchester, UK). The mass spectrometer was operated in both positive and negative electrospray ionization mode. A mass range of *m*/*z* 50−1200 was used. The source and IMS parameters are summarized in Table S2. Leucine enkephalin was used as the lock mass [M + H]^+^ at *m*/*z* 556.2771 and [M-H]^−^ at *m*/*z* 554.26202. Sodium formate solution was used for external instrument calibration. The Synapt G2 HDMS was equipped with a traveling wave “Triwave™” geometry, including an ion mobility cell (IMS T-wave). The TOF analyzer was operated in the V resolution mode with an average mass resolution of m/Δm 20,000 (full-width at half-maximum definition). Data acquisition of an ion mobility experiment consisted of 200 bins. The cross-collision section (CCS) values, obtained in nitrogen, were experimentally determined using singly charged Poly-DL-alanine oligomers as the TWIM calibrant species for ESI+. The CCS values were derived according to previously reported procedures [46] with the following general equation ln(CCS’) = xln(dt’) + ln(A). Calibration values and curves are shown in the Supplementary Materials (Table S3). The ion mobility resolution was ~40 Ω/ΔΩ (fwhm). The N_2_ CCS values reported were determined at the apex of the ion-mobility peak.

#### 2.3.5. Raw Data Processing

All LC-IM–MS raw data files data processing, peak detection, and peak matching across samples using retention time (t_R_) correction and chromatographic alignment along with drift time were performed using Progenesis QI (Waters MS Technologies, Manchester, UK) to yield a data matrix containing retention times, accurate masses, drift time, and peak intensities. Experimental CCS were determined, as stated above, to include CCS instead of drift time values in the data matrix. CCS errors between experimental and theoretical values (%) were then calculated from the LipidsCCS Web Server (Zhu Lab) [47]. The preprocessing step resulted in an X-matrix where tR, CCS, and *m*/*z* values were concatenated into “t_R_ _m/z_CCS” features (in columns) present in each sample (in rows) with corresponding peak areas.

#### 2.3.6. Quality Control

Aliquoted 10 μL extracts were mixed to generate pooled quality control samples (QCs). The QCs and solvent blank samples (mobile phase) were injected sequentially in-between the plasma samples. In addition, a dilution series of QC samples (1:2, 1:4, 1:8, and 1:16 original concentration) were used to assess the quality of the extracted features. In this study, we used a filter strategy in which the features’ intensity must be correlated to the matrix concentrations in a series of diluted QC samples in order to be included in further analysis. Feature groups with a correlation coefficient of less than 0.70 were removed from the dataset. Furthermore, datasets were refined by the removal of feature groups that did not meet the threshold of peak area measurement precision prior to data analysis. This approach used RSD values derived from repeated measurements of a pooled QC sample. The threshold was set to RSD < 25% to enhance the biological interpretation of metabolomics data.

### 2.4. Tissue Metabolic Phenotyping

#### 2.4.1. Reagents

MALDI matrices, including 2.5-dihydroxybenzoic acid (DHB) and 9-aminoacridine hydrochloride monohydrate (9-AA), were purchased from Sigma-Aldrich (St. Louis, MO, USA). Sodium trifluoroacetate (NaTFA, Sigma–Aldrich) at 0.1 mg mL^−1^ (ACN/H_2_O 50:50 [*v*/*v*]) was used for external calibration before each analysis.

#### 2.4.2. Sample Preparation

Fresh frozen 10-µm sections of human brain tissue were mounted on conductive ITO-coated glass slides 75 × 25 mm (Bruker, Bremen, Germany) and stored at −80 °C until analysis. Matrix solutions were applied with an automatic micro sprayer HTX TM-Sprayer (HTX Imaging, Chapel Hill, NC, USA) as previously described [31]. In the positive-ion mode, the DHB matrix (30 mg mL^−1^ in MeOH/H_2_O 50:50 [*v*/*v*]) was deposited with the following parameters: nozzle temperature 80 °C, nozzle velocity 1200 mm min^−1^, N_2_ pressure 10 psi, N_2_ flow rate 2 L min^−1^, number of passes 12, flow rate 100 µL min^−1^, and track spacing 3 mm. In negative-ion mode, 9-AA matrix (10 mg mL^−1^, EtOH/H_2_O 70:30 [*v*/*v*]) was sprayed using the following parameters: nozzle temperature 90 °C, nozzle velocity 1200 mm min^−1^, N_2_ pressure 10 psi, N_2_ flow rate 3 L min^−1^, number of passes 2, flow rate 120 µl min^−1^, track spacing 3 mm, and drying time 30 s between passes. Each slide was vacuum-dried before analysis.

#### 2.4.3. Data Acquisition and Data Processing

Data were acquired on a FTICR instrument (SolariX XR, Bruker, Bremen, Germany) equipped with a 12-Tesla superconducting magnet and a dynamically harmonized ICR cell. This instrument was also equipped with both a laser desorption ionization source (Smartbeam II, Nd:YAG × 3 laser at 355 nm, Bruker, Bremen, Germany) and an electrospray (ESI) source. Each MALDI spectrum for each position was the result of 1 scan and 500 consecutive laser shots. Spectra were acquired over a 100 µm raster. Before imaging analyses, the instrument was externally calibrated in the required mode by NaTFA infusion via an ESI source, then internally calibrated by assigning known metabolites from *m*/*z* 150–1000 via MALDI source. Thus, the instrument was auto calibrated during image acquisition. In positive-ionization mode, calibration was performed by assigning C_7_H_6_O_4_ (*m*/*z* 155.033885 [M+H]^+^, matrix peak), C_7_H_15_NO_3_ (*m*/*z* 162.112470 [M+H]^+^, carnitine), C_7_H_6_O_4_ (*m*/*z* 177.015829, [M+Na]^+^, matrix peak), C_5_H_14_NO_4_P (*m*/*z* 184.073321, [M+H]^+^, phosphocholine), C_9_H_17_NO_4_ (*m*/*z* 204.123034, [M+H]^+^, acetyl carnitine), C_14_H_8_O_6_ (*m*/*z* 273.039364, [M+H]^+^, matrix peak), C_21_H_12_O_9_ (*m*/*z* 409.055408, [M+H]^+^, matrix peak), C_40_H_80_NO_8_P (*m*/*z* 734.569432, [M+H]^+^, lipid), and C_42_H_82_NO_8_P (*m*/*z* 798.54096, [M+K]^+^, lipid). In the negative-ion mode, the assigned peaks were C_13_H_10_N_2_ (*m*/*z* 193.077122, [M−H]^−^, matrix peak), C_10_H_15_N_5_O_10_P_2_ (*m*/*z* 426.022139, [M-H]^−^, adenosine diphosphate), C_10_H_16_N_5_O_13_P_3_ (*m*/*z* 505.988470, [M−H]^−^, adenosine triphosphate), C_41_H_80_NO_8_P (*m*/*z* 744.554879, [M−H]^−^), and C_47_H_83_O_13_P (*m*/*z* 885.549853, [M−H]^−^). Data size was set at 2 million points for a transient length of 0.87 s, and spectra were acquired with a 97% data file reduction. A single MSI measurement was performed for each specimen. Images were captured using FTMS control and FlexImaging (v 5.0, Bruker) software. Images were processed with SCiLS Lab Pro software (Bruker Daltonics, Bremen, Germany). The total ion current method was used for normalization, and *m*/*z* intervals were automatically set at ±1 ppm. Images were viewed using both FlexImaging and SCiLS Lab software (Bruker Daltonics, Bremen, Germany).

### 2.5. Metabolite Annotation and Identification

Preliminary assignments based on accurate mass measurements were performed using the mass spectrometry databases, METLIN [48] and HMDB [49], using a threshold of ±2 ppm. For some metabolites, the precise raw formula led to one hit. For CCS-based annotations, the AllCCS database was used [50]. Only positive ion mode highlighted discriminative ions. Other ions were identified via “on-tissue” tandem mass spectrometry experiments using MALDI tandem MS/MS. Ions of interest were first isolated using a window of ± 1 Da, then fragmented by collision-induced dissociation with energy levels between 10 and 40 eV. For each MS/MS analysis, 50 scans were accumulated for better sensitivity. Spectra were reprocessed using Data Analysis 4.4 software (Bruker Daltonics, Bremen, Germany) and recalibrated with the single-point calibration option. The full list of annotated features is presented in Tables S4 and S5.

### 2.6. Circulating Cell-Free DNA Acquisition

Circulating cell-free DNA was extracted from blood samples right before surgery and as previously described [51]. Briefly, six milliliters of whole blood was collected in tubes containing ethylenediaminetetraacetic acid (EDTA). Within two hours after blood collection, the tubes were centrifuged; the plasma was then extracted and stored at −80 °C until use. cfDNA was extracted from 1 mL to 5 mL of plasma using the QIAamp Circulating Nucleic Acid kit^®^ (Qiagen, Hilden, Germany) according to the manufacturer’s instructions. The sample was eluted in a final volume of 30 µL and stored at −20 °C. Double-stranded DNA quantification was performed by a fluorometric method using a Quantit™ PicoGreen® dsDNA Assay kit (Invitrogen, Carlsbad, CA, USA) and a Twinkle LB970 microplate fluorimeter (Berthold, Bad Wildbad, Germany). For each sample, cfDNA quantification was performed in duplicate from 2 µL of eluate, and normalization was performed using a standard calibration curve of known concentrations of standard dsDNA (from 0 to 10 ng). Overall, cfDNA concentration reflects both cell-free genomic DNA as well as circulating tumor DNA (ctDNA).

### 2.7. Data Analysis

Ion intensities or absolute concentrations were log-transformed and Pareto-scaled [52]. Missing values were imputed using the nearest neighbor averaging algorithm using the impute.knn function in the impute R package. The full data matrices are presented in Tables S6–S8. Univariate analyses were performed using *t*-tests or Mann–Whitney U depending on the normal distribution of the data. The Limma package [53] was used for differential analysis using sex, and age was taken into account by adding it as a covariate. Spearman correlation analysis was performed using R software. Euclidean distance was used as a similarity measure in the clustering analysis. Principal Component Analysis was used as a dimension reduction technique using log-transformed and Pareto-scaled datasets. False discovery rates were corrected using the Benjamini–Hochberg–Yekutieli method [54], and *p* < 0.05 was considered statistically significant. For network analysis, the first step was to compute several glasso-based networks (GLN) [55]. Three kinds of GLNs were calculated; control + disease samples, control samples only, and all the combinations of samples, including control + “disease-minus-one-patient” to get patient-specific networks. Networks were then constructed from each GLNs data matrix and pruned with each other to get specific networks. The idea of network pruning is to remove edges in a general network that are also found in a more specific network. So, we pruned the “disease + control” network with the “control” network in order to keep only the edges that were disease-specific. Thus, this step resulted in a “disease-specific” network. This step was done using the CTD R package [56]. Using the same strategy, networks of controls + “disease-minus-one” samples were pruned with the controls samples network to obtain a “disease-specific-minus-one-patient” network. This network was then pruned with the “disease-specific” network calculated above in order to extract “patient-specific” metabolic signatures. A summary overview of the network strategy is presented in Figure S1. The metabolites present in more than 50% of these “patient-specific” networks were selected to build a Consensus-Network, enabling the visualization of key metabolic signatures for the disease. To test the discriminatory power of this signature, Random Forest models were tuned for every possible combination of metabolites from the Consensus-Network. Random Forest models were built using the ranger package [57] and the caret package in R [58]. The models were tuned over ~50 repeats to obtain robust classification probabilities. Performances of the models were assessed with the MLeval package in R. The main metric for predictive performance assessment was the area under the curve (AUC) for the resulting receiver operating characteristic (ROC) curve. All analyses were done using R software [59]. All data generated or analyzed during this study are included in this published article and its Supplementary Materials.

## 3. Results 

### 3.1. Cohort Description

**Figure 1:**
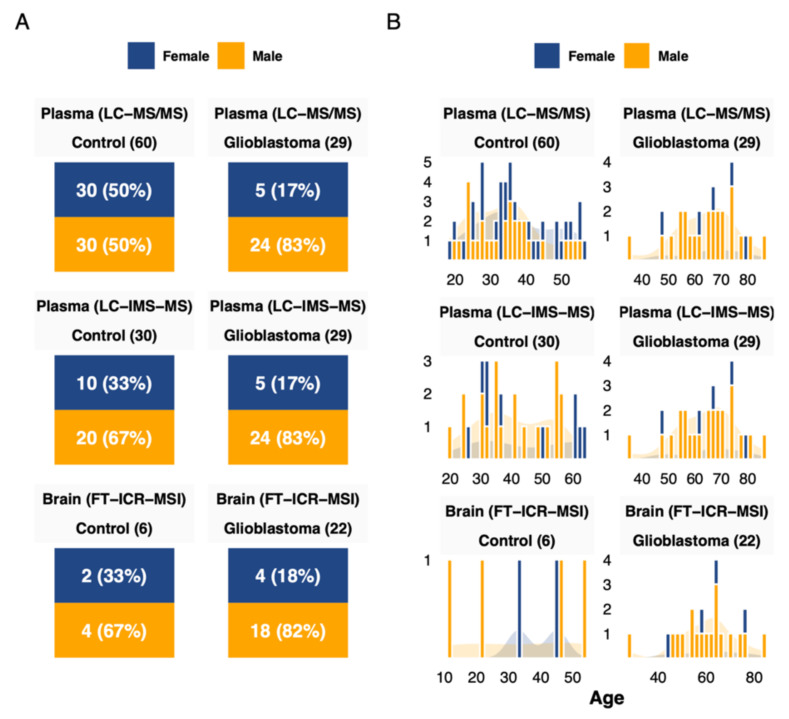
Cohort overview. (**A**) Cohort description and sex distribution related to each dataset. (**B**) Age distribution by sex and dataset.

The mean glioblastoma showed a male predominance of 82%. The mean age at diagnosis was 66 ± 11.4 years for females and 64 ± 11.1 years for males. Regarding the performed surgery, it consisted of a biopsy in 56.7% and resection in the other cases. The mean controls age was 39 ± 12.5 years for females and 37 ± 10.9 years for males. A cohort overview is presented in Figure 1, and a summary is presented in Table 1. The detailed clinical and biological data are listed in Table S9. Three different metabolomics methods were used to explore metabolic profile differences between glioblastoma and control samples. They included untargeted mass spectrometry imaging on the tissue samples and both targeted and untargeted analysis on plasma samples. The full list of analyzed metabolites is presented in Tables S4 and S5, and the full data matrices are presented in Tables S6–S8.

### 3.2. Unsupervized Exploratory Analysis

**Figure 2:**
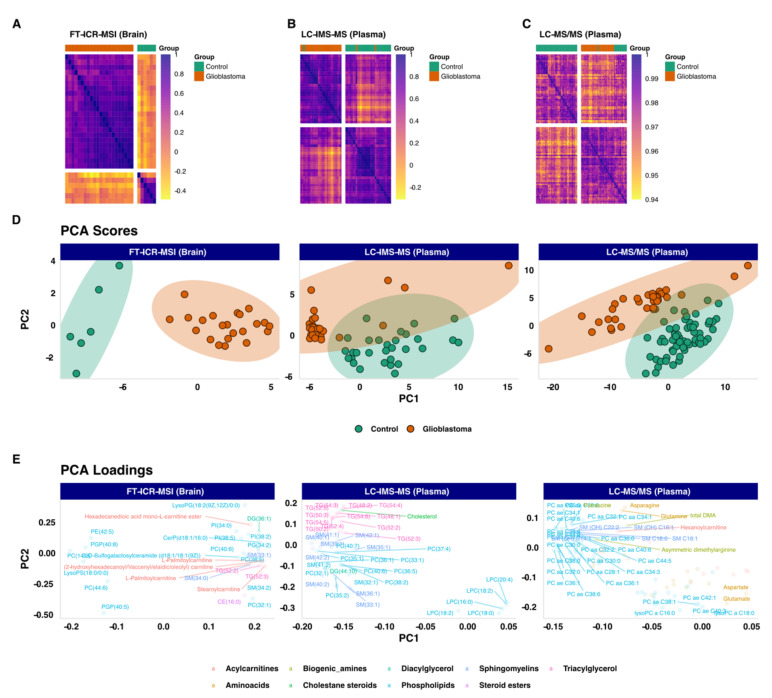
Unsupervised exploratory analysis overview. (**A**) Heatmap of the sample’s spearman correlation analysis based on untargeted FT-ICR-MS data. (**B**) Heatmap of the sample’s spearman correlation analysis based on untargeted LC-IMS-MS data. (**C**) Heatmap of the sample’s spearman correlation analysis based on targeted LC-MS/MS data. (**D**) Principal Component Analysis scores plot based on untargeted FT-ICR-MS (Explained variance: PC1 = 65%, PC2 = 7%), untargeted LC-IMS-MS (Explained variance: PC1 = 55%, PC2 = 15%) and targeted LC-MS/MS (Explained variance: PC1 = 32%, PC2 = 16%) datasets, respectively. (**E**) Principal Component Analysis loadings plot based on untargeted FT-ICR-MS (Explained variance: PC1 = 65%, PC2 = 7%), untargeted LC-IMS-MS (Explained variance: PC1 = 55%, PC2 = 15%) and targeted LC-MS/MS (Explained variance: PC1 = 32%, PC2 = 16%) datasets, respectively.

To analyze the data, an exploratory approach was first used. This unsupervised analysis aimed to track samples’ clustering trends based on their underlying metabolic profiles. We unveiled these grouping trends using Spearman correlations and hierarchical clustering of the samples. The results showed a clear separation of two distinct groups related mainly to control and GBM samples Figure 2A–C. This was observed in all three datasets (MSI, untargeted, or targeted plasma metabolomics) as shown in the heatmaps in Figure 2A–C. Higher-resolution heatmaps are presented in Figure S2, and full correlation matrices are presented in Tables S10–S12. The same clustering trends were also observed using the PCA scores plots, Figure 2D, that showed two clear groups related to GBM and control samples. This separation was mainly observed on the PC1 dimension. The PCA scores matrices are presented in Table S13.

### 3.3. Differential Expression Analysis

**Figure 3:**
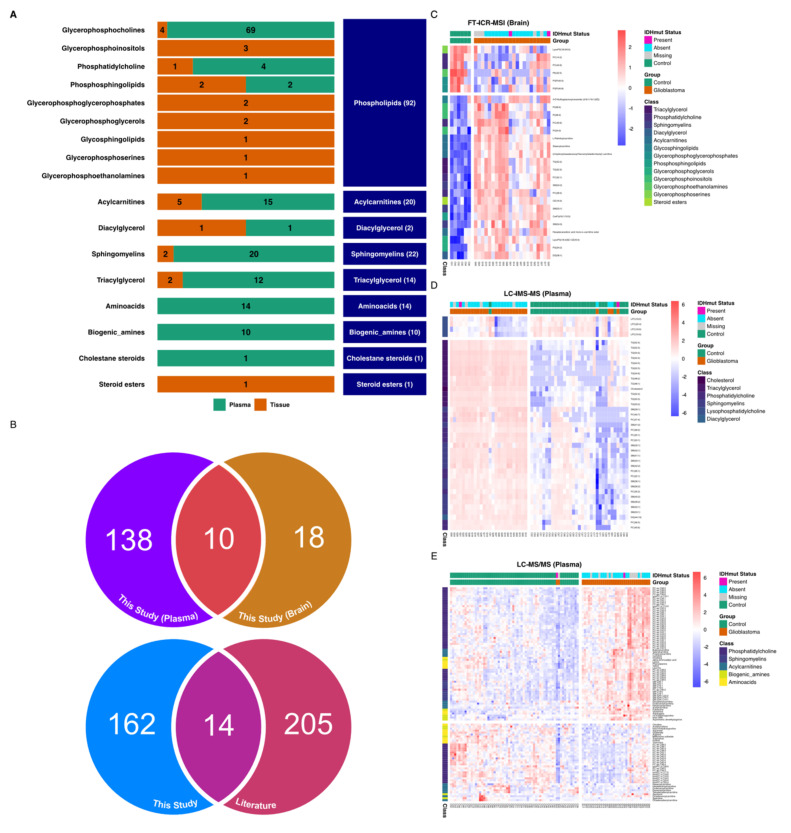
Differential analysis overview. (**A**) Differential analysis results between control and glioblastoma samples highlighting metabolic classes. One hundred and seventy-six metabolites were identified based on an adjusted *p*-value of 0.05. One hundred and forty-eight plasma-based metabolites and 28 Tissue-based metabolites. (**B**) Upper: Ven diagram representation of the overlap between the identified differentially expressed metabolites in Plasma versus Brain. Lower: Ven diagram representation of the overlap between the identified differentially expressed metabolites in this study versus reported human metabolomics-based GBM reported literature. (**C**) Heatmap showing the relative levels of statistically differential metabolites in different patients’ tissues using FT-ICR-MS data. (**D**) Heatmap showing the relative levels of statistically differential metabolites in different patients’ plasma using LC-IMS/MS data. (**E**) Heatmap showing the relative levels of statistically differential metabolites in different patients’ plasma using LC-MS/MS data. Detailed results are presented in Table S13.

**Figure 4:**
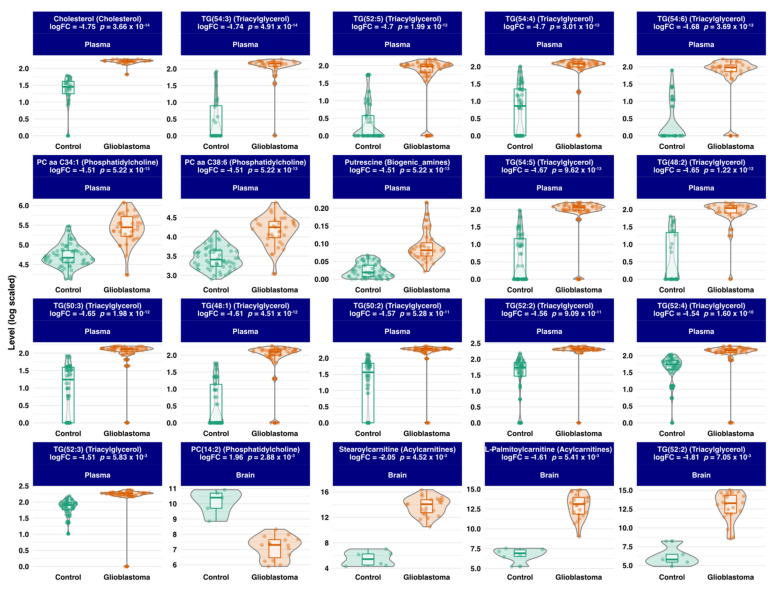
Boxplots of the top (based on adjusted *p*-values) twenty newly reported metabolites and related biological matrix.

To go further in the analysis, we performed a differential analysis between the two groups GBM versus Control, using each of the three datasets. The analysis identified 176 annotated metabolites that were differentially expressed between GBM and control samples. The plasma-based comparison yielded 148 metabolites, including 75 phospholipids (69 glycerophosphocholines, 4 phosphatidylcholines, and 2 phosphosphingolipids), 20 sphingomyelins, 15 acylcarnitines, 14 amino acids, 12 triacylglycerids, 10 biogenic amines, 1 cholestan steroid, and 1 diacylglycerol. The tissue-based comparison unveiled 28 metabolites, including 17 phospholipids (4 glycerophosphocholines, 3 phosphoinositols, 1 phosphatidylcholine, 2 phosphosphingolipids, 2 glycerophosphoglycerophaosphate, 2 glycerophosphoglycerid, 1 glycosphyngolipid, 1 glycerosphingoserine, and 1 glycerophosphoethanolamine), 5 acylcarnitines, 1 diacylglycerol, 2 sphingomyelins, 2 triacylglycerols, and 1 steroid ester. The summary results are presented in Figure 3A. The list of metabolites and their statistics-related metrics is presented in Table S14. Furthermore, we compared the retrieved metabolites with previously reported human GBM metabolomics-based literature [38,39,40,41,60]. The summary results are presented in Figure 3B, and the full results are presented in Table S15. The overlap with previously reported metabolites includes 4 biogenic amines (Putrescine, cis-4-Hydroxyproline, trans-4-Hydroxyproline, Spermine), 8 amino acids (Glutamine, Asparagine, Ornithine, Lysine, Tryptophan, Citrulline, Threonine, Valine), 1 steroid ester (CE(22:6)) and Carnitine. The related boxplots and correlations are presented in Figures S3 and S4, respectively. In contrast, the metabolites that were not reported in the above-mentioned literature include mainly Acylcarnitines, Biogenic amines, Cholesterol, Phosphatidylcholines, and Triacylglycerols. Based on adjusted *p*-values, the top metabolites are presented in Figure 4, and their Spearman correlations are presented in Figure S5.

### 3.4. Correlation Analysis

**Figure 5:**
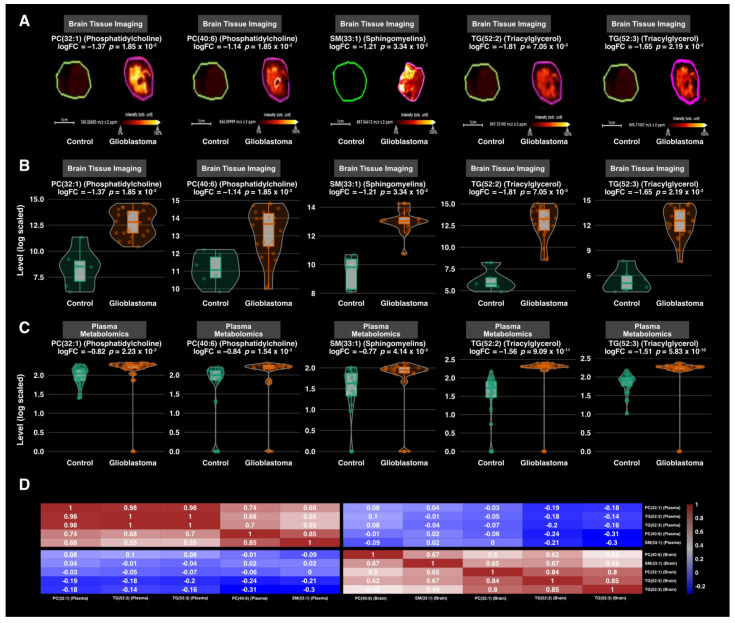
Overlap analysis between tissue-based and plasma-based metabolomics results. (**A**) Brain tissue section distribution of selected overlap lipids. Ion images were generated using SCiLs Lab software. (**B**) Brain tissue sections boxplots of the selected top five differentially expressed metabolites among the overlap lipids with related adjusted *p*-values. The *y*-axis shows the log-scaled average intensity. (**C**) Plasma boxplots of the selected top five differentially expressed lipids among the overlap metabolites with related adjusted *p*-values. The *y*-axis shows the log-scaled intensity. (**D**) Spearman correlation analysis between the selected top five lipids that overlap between tissue-based and plasma-based metabolomics analysis.

Given the opportunity to have in-tissue metabolomics with matched-plasma samples, we compared the plasma-based and tissue-based metabolic profiles and found ten lipids that overlap between the two lists. These include, phosphatidylcholines LPC(18:0), PC(36:5), PC(40:6), PC(32:1), sphingomyelins SM(33:1), SM(36:2) and triacylglycerides TG(56:7), TG(52:2), TG(52:4), TG(52:3). The boxplots of the top five lipids are presented in Figure 5. They include PC(40:6), PC(32:1), SM(33:1), TG(52:2), and TG(52:3). We also evaluated these lipid intercorrelations between tissue samples and their matched plasma samples. The figure did not show a high correlation between plasma and brain levels. However, the heatmap showed a high intra-matrix correlation. The top metabolites (abs(log fold change > 1.5) and adjusted *p*-value < 0.01) are presented in Figure 5. Furthermore, we explored the overall intra-GBM group variability using the coefficient of variation as a proxy in both tissue and plasma samples. The results showed high inter-tissue variability (CV median = 127 %) compared to plasma (CV median = 36%) (Figure S6A,B). This highlights an inter-tumor metabolic heterogeneity. We also explored the variability of the above-mentioned overlap lipids that clearly showed the same observation (Figure S6C). The full metabolite correlation matrix is presented in Table S16.

**Figure 6:**
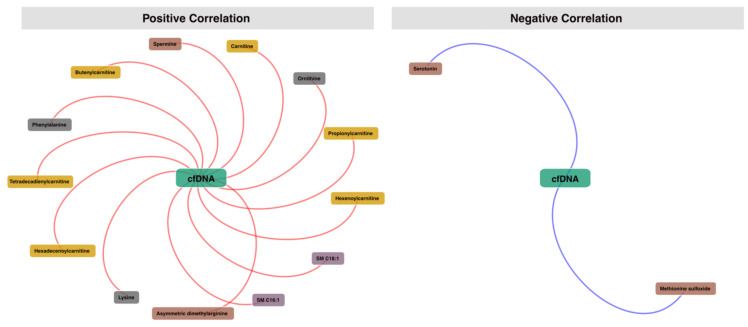
Spearman correlation analysis between metabolic profiles and free circulating DNA. Cut-off was set at adjusted *p*-value > 0.05 and abs(Spearman rho) > 0.25. Multiple test correction was applied for *p*-values using Benjamini and Hochberg method is used.

Given the rising interest in using circulating free DNA (cfDNA) in oncology [51], we also assessed the associations between the retrieved metabolic profile and cfDNA using Spearman correlations. The analysis yielded 13 positive correlations and 2 negative correlations with a cut-off set at adjusted *p*-value < 0.05 and abs(Spearman rho) > 0.25. The positively correlated metabolites included Ornithine (Spearman rho = 0.52), Lysine (Spearman rho = 0.49), Phenylalanine (Spearman rho = 0.46), Propionylcarnitine (Spearman rho = 0.45), Carnitine (Spearman rho = 0.45), Hexadecenoylcarnitine (Spearman rho = 0.42), Tetradecadienylcarnitine (Spearman rho = 0.4), Hexenoylcarnitine (Spearman rho = 0.35), Spermine (Spearman rho = 0.3), SM C18:1 (Spearman rho = 0.28), Butenylcarnitine (Spearman rho = 0.29), SM C16:1 (Spearman rho = 0.29), and Asymmetric dimethylarginine (Spearman rho = 0.27). The negatively correlated metabolites included two biogenic amines Serotonin (Spearman rho = −0.68) and Methionine sulfoxide (Spearman rho = −0.29). A network visualization is presented in Figure 6, and full results are presented in Table S17. The full heatmap correlation matrix is presented in Figure S7.

### 3.5. Network and Predictive Machine Learning Analysis

**Figure 7:**
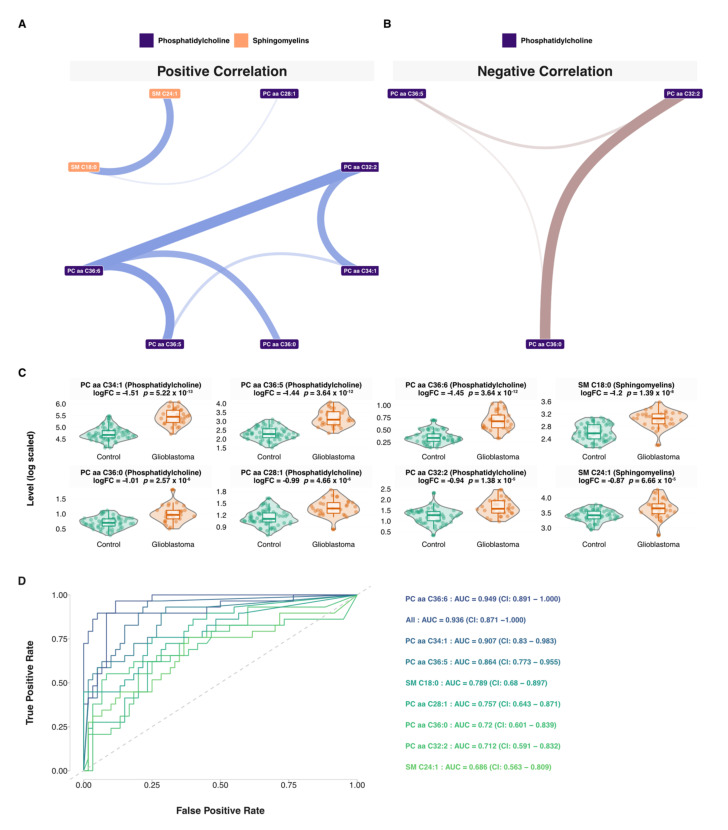
Network and machine learning analysis. (**A**) Negative correlation network visualization of the glioblastoma consensus metabolic signature. (**B**) Positive correlation network visualization of the glioblastoma consensus metabolic signature. Line width is proportional to the Spearman correlation. (**C**) Boxplots of the consensus plasma metabolic signature. (**D**) ROC curves of the Random Forest predictive models, including seven phosphatidylcholines (PC aa C36:6, PC aa C32:2, PC aa C36:0, PC aa C36:5, PC aa C34:1, and PC aa C28:1) and two sphingomyelins (SM C18:0 and SM C24:1). AUC and 95% confidence intervals (CI) were calculated using balanced subsampling with 50 repeats.

Using the targeted plasma metabolomic dataset, different correlation networks were built using control, disease, or both samples. Using these correlation networks, 29 patient-specific metabolic signatures were extracted (Figures S8–S36, Tables S18–S20). These patient-specific metabolic networks highlighted the metabolic individuality of each patient. Based-on this, we explored the similarity of these signatures between patients. The results revealed 69 unique metabolites spanning phosphatidylcholines (57%), amino acids (14%), acylcarnitines (12%), sphingomyelins (12%), and biogenic amines (6%). Eleven metabolites were found in only one signature and included aspartate, proline, valine, hexanoyl-carnitine, SM C16:0, lysoPC a C16:0, PC aa C42:1, PC aa C42:4, PC aa C42:5, PC ae C32:2, PC ae C34:0, PC ae C38:6. Two patients with mutated IDH status, PGB_04 and PGB_24, both males at 35 and 45 years old, respectively, yielded the following signatures. For PGB_04 the signature included 11 metabolites; 6 phosphatidylcholines, 3 acylcarnitines, and 2 sphingomyelins (Figure S11) and for PGB_024 male at 35 years old, the signature included 18 metabolites; 14 phosphatidylcholines, 1 acylcarnitine, 1 biogenic amine, and 2 sphingomyelins (Figure S31). Then, we identified the most redundant metabolites found in more than 50% of the patients’ signatures which included eight metabolites, seven phosphatidylcholines (PC aa C28:1, PC aa C32:2, PC aa C34:1, PC aa C36:0, PC aa C36:5, PC aa C36:6), and two sphingomyelins (SM C18:0, SM C24:1) (Figure 7A,B, Table S21). The related boxplots are presented in Figure 7C. Based on this signature, we explored the predictive performance of each lipid and all its combinations using predictive machine learning. We built Random Forest predictive models based on each lipid alone as well all possible combinations of the eight lipids. Area under curve and ROC curves were used as performance metrics (Table S22). The eight univariate models and their combinations are shown in Figure 7C. These models included PC aa C36:6 (AUC = 0.949: 0.891–1.000), PC aa C34:1 (AUC = 0.907: 0.83–0.983), PC aa C36:5 (AUC = 0.864: 0.773–0.955), SM C18:0 (AUC = 0.789: 0.68–0.897), PC aa C28:1 (AUC = 0.757: 0.643–0.871), PC aa C36:0 (AUC = 0.72: 0.601–0.839), PC aa C32:2 (AUC = 0.712: 0.591–0.832), SM C24:1 (AUC = 0.686: 0.563–0.809). All included lipids models showed an AUC = 0.936: 0.871–1.000. It is worth mentioning that the most predictive model was the PC aa C36:6 (AUC = 0.949: 0.891–1.000). The full list of investigated models is presented in Table S23.

## 4. Discussion

Despite a sustained research effort in the fight against glioblastoma, the prognosis of this condition remains devastating, and its metabolic signaling pathways are barely understood. To tackle this challenge, omics-based technologies could shed light on this aggressive disease. Metabolic phenotyping through metabolomics is one of the promising tools to interrogate functional metabolic readouts.

In this study, we identified four metabolites’ classes as proxies of deep metabolic remodeling in plasma and tissue samples from glioblastoma patients, which included phospholipids, acylcarnitines, sphingomyelins, and Triacylglycerols. Some of the highlighted metabolites in our study have been previously reported. This is the case for a number of amino acids, such as asparagine, glutamine, lysine, citrulline, or valine, for example (Figure 3) [38,39,41]. Indeed, amino acid metabolism is an area of interest in the race for new therapies for glioblastoma. The discovery of the Isocitrate Dehydrogenase (IDH) metabolic pathway has revived this antimetabolic approach, which aims at reducing the synthesis of these molecules, which play an essential role in tumor development and progression [61]. Recently differences in the levels of certain amino acids in the blood of patients with glioblastoma compared to healthy subjects have been demonstrated [62]. These data empower the central role of amino acids in the genesis of glioblastoma. In parallel to amino acid metabolism, our study also highlighted the role of lipids in this disease. Glycerophosphocholines (GPC) were one key class highlighted in this study. Most prominent GPC were PC aa C34:1, PC aa C38:6, PC (14:2), PC aa C36:5, lysoPC a C18:0, PC aa C28:1, PC aa C32:2, PC aa C36:0, and PC aa C36:6 (Figure 3A, Figure 4, Figure 5 and Figure 7). GPC are precursors lipids of acetylcholine [63]. Cholines are important membrane components and known as markers of tumor progression [64]. A choline peak visualized by MRI spectroscopy is a diagnostic criterion for glioblastoma that has been used in clinical practice for several years [38]. Similarly, it has been shown that choline blood levels in glioblastoma patients are correlated with tumor progression [65]. More recently, following in-vivo brain micro dialysis studies [66], it appears that choline levels in the peri-tumor tissue also correlate with its degree of invasion. One of the pathophysiological hypotheses is that glioblastoma modifies its environment to make it favorable to its development and proliferation [67]. Furthermore, one of the key elements for glioblastoma progression is energy substrate availability. The cerebral high lipid enrichment helps deliver the energy necessary for tumor growth [68]. Unlike normal cells, which deliver energy from mitochondrial phosphorylation, tumoral glial cells get their energy from glycolysis and have intense lipidogenesis. Taïb et al. [69] showed that oleic acid, a monounsaturated fatty acid, increases triacylglycerol production in malignant glial cells but not in normal glial cells, arguing for a metabolic reprogramming of the malignant glial cell environment to allow them to spread [70]. Sphingolipids are also involved in this phenomenon and were highlighted in our analysis through SM (0H) C22:2, SM (0H) C16:1, SM C18:0, and SM C18:1 and SM C24:1 (Figure 3A, Figure 4, Figure 5, and Figure 7). These lipids are abundant in the brain and are essential membrane constituents highly expressed in glial cells. Alterations of the sphingolipid pathway resulting in lower levels of ceramides which have pro-apoptotic properties, are thought to play a role in malignant cell dissemination, and malignant glial cells have also been reported to evade apoptosis by converting ceramides to sphingosine-1-phosphate, thus preventing apoptosis [71]. Thus, the action of oncological treatments of glioblastoma, which generate sphingomyelinases to lyse sphingomyelins into ceramides, escape this cell death phenomenon [67]. The exact drivers of this process are not yet well understood. Steroylcarnitine and L-palmitoylcarnitine, which belong to the Acylcarnitines (AC) class, are also modulated during glioma genesis (Figure 3A and Figure 5). AC primary function is to allow fatty acids transport to feed mitochondrial β-oxidation and thus ATP production. Based on a recent study on patient-derived xenograft models of GBM, it appears that AC are abundantly found within and at the edge of these tumors, and this may provide a fertile ground for glioblastoma spread [72]. These advances on how glioblastoma modulates its biological environment to promote its spread, notably through lipid metabolism, opens, obviously, promising ways to new targeted therapeutic strategies and biomarker discovery. Interestingly, this study showed that circulating free DNA level was positively correlated with sphingomyelins (SM C16:1 and SM C18:1) and Acylcarnitines (Figure 6), which are major substrates of tumor progression by providing the glioblastoma with the energy. Lipid droplets (LDs) are organelles that are commonly found in fatty tissues storing lipids, triglycerol, and cholesterol esters. Recently, the presence of LDs has been demonstrated in glial and glioblastoma cell cultures in vivo [73]. These LDs are not only a simple reservoir of fatty acids but have an impact on important cell processes such as cell cycles, cell migration of glial cells, and their resistance to apoptosis and conventional treatments [69]. LDs seem to be promising biomarkers to probe tumor progression [74]. It has been established for a long time [75] that GBM diverts brain lipid metabolism to its advantage to sustain the energy stores needed for their own expansion. By focusing on these LDs, it has been shown that they play a central role in this lipid metabolism reprogramming [73]. Furthermore, it has been reported that autophagy-mediated hydrolysis of these LD maintains energy homeostasis [76]. Thus, limiting the access of glioblastoma to this lipid stock represents a potentially innovative treatment avenue. Despite intensive chemoradiotherapy, the median survival of patients with GBM remains at around 15 months. In recent years, lipidomics has opened the way to new perspectives [77]. The goal is to prevent glial cells from spreading by modulating their environment. Sphingolipids are one of these targets by promoting the formation of apoptosis-inducing ceramides. The action of temozolomide, the first-line chemotherapy for glioblastoma, on the biological environment of glial cells is being actively studied. Temozolomide modifies the extracellular vesicles both in size and content [78]. These extracellular vesicles release fatty acids and proteins that modulate the action of tumor-associated macrophages [78]. Chemotherapy may thus have a modulating effect on the environment of glial cells via these vesicles. The macrophages associated with tumors are major components of tumor spread. Thus, targeting these macrophages could counteract the chemo- and radio resistance of glial cells. As lipids are ubiquitous in the brain, they have been used as vectors for targeted therapies using CRISPR-Cas9 technology [79]. Based on the seminal paper by Garofano et al. [80], a classification of glioblastomas based on cell signaling pathways has revealed a therapeutic sensitivity in the mitochondrial glioblastoma subgroup. These preliminary results, although encouraging, need to be validated and replicated in humans. Some limitations have to be noted regarding our study. The sample size was limited for the tissue-based metabolomics which is mainly related to practical constraints and patients’ recruitment. Larger cohorts are needed to confirm the present results, ideally, including other bulk and/or single-cell panomics to have a broader view of the impaired biological pathways and their clinical effect.

## 5. Conclusions

In summary, our study demonstrated the potential of systems-based metabolomics strategies to holistically interrogate biological plasticity in glioblastoma and parse the role of lipids in tumor progression by the modulation of its own environment. The next step of our work would be the study of therapeutic modulations of these metabolic signatures in order to probe treatment effectiveness. Applying these plasma metabolic patterns could also inform clinical practice to adapt treatment before MRI or clinical modification. A better understanding of metabolic impairments underlying glioblastoma spread would also drive the development of targeted therapies to prevent glioblastoma from promoting its own extension to the surrounding tissues. Such systems-based strategies highlight the importance of multi-omics and multimodal investigations to understand glial lesions to pave the way to more personalized therapies and, ultimately, achieve the promise of precision medicine.

## Figures and Tables

**Table 1:** Main cohort summary.

Characteristic	Control (Brain), *n* = 6 ^1^	Control (Plasma), *n* = 60 ^1^	Glioblastoma, *n* = 22 ^1^	*p*-Value ^2^
Sex				0.023
Female	2 (33%)	30 (50%)	4 (18%)	
Male	4 (67%)	30 (50%)	18 (82%)	
Age (Years)	39 (25, 46)	34 (27, 42)	62 (54, 66)	<0.001
ATRX_mutation				
Absent			1 (6.2%)	
Present			15 (94%)	
Unknown			6	
IDH_mutation				
Absent			15 (88%)	
Present			2 (12%)	
Unknown			5	

^1^ n (%); Median (IQR), IQR: Interquantile range; ^2^ Fisher’s exact test; Kruskal–Wallis rank-sum test.

## Data Availability

Data supporting the finding are presented in the text and Supplementary Material.

## Supplementary Materials

The following are available online at www.mdpi.com/article/10.3390/cancers13205157/s1, Figure S1: Network analysis overview. Networks are built using Partial Correlation, Figure S2: Spearman correlations between samples, Figure S3: Boxplots of previously reported metabolites, Figure S4: Heatmap of Spearman correlations of previously reported metabolites, Figure S5: Heatmap of Spearman correlations of newly reported metabolites, Figure S6: Intra-group (GBM) variation, Figure S7: Heatmap of Spearman correlations between all significant metabolites, Figure S8: Network-based patient specific plasma metabolic signature (Patient PGB_01), Figure S9: Network-based patient specific plasma metabolic signature (Patient PGB_02), Figure S10: Network-based patient specific plasma metabolic signature (Patient PGB_03), Figure S11: Network-based patient specific plasma metabolic signature (Patient PGB_04), Figure S12: Network-based patient specific plasma metabolic signature (Patient PGB_05), Figure S13: Network-based patient specific plasma metabolic signature (Patient PGB_06), Figure S14: Network-based patient specific plasma metabolic signature (Patient PGB_07), Figure S15: Network-based patient specific plasma metabolic signature (Patient PGB_08), Figure S16: Network-based patient specific plasma metabolic signature (Patient PGB_09), Figure S17: Network-based patient specific plasma metabolic signature (Patient PGB_10), Figure S18: Network-based patient specific plasma metabolic signature (Patient PGB_11), Figure S19: Network-based patient specific plasma metabolic signature (Patient PGB_12), Figure S20: Network-based patient specific plasma metabolic signature (Patient PGB_13), Figure S21: Network-based patient specific plasma metabolic signature (Patient PGB_14), Figure S22: Network-based patient specific plasma metabolic signature (Patient PGB_15), Figure S23: Network-based patient specific plasma metabolic signature (Patient PGB_16), Figure S24: Network-based patient specific plasma metabolic signature (Patient PGB_17), Figure S25: Network-based patient specific plasma metabolic signature (Patient PGB_18), Figure S26: Network-based patient specific plasma metabolic signature (Patient PGB_19), Figure S27: Network-based patient specific plasma metabolic signature (Patient PGB_20), Figure S28: Network-based patient specific plasma metabolic signature (Patient PGB_21), Figure S29: Network-based patient specific plasma metabolic signature (Patient PGB_22), Figure S30: Network-based patient specific plasma metabolic signature (Patient PGB_23), Figure S31: Network-based patient specific plasma metabolic signature (Patient PGB_24), Figure S32: Network-based patient specific plasma metabolic signature (Patient PGB_25), Figure S33: Network-based patient specific plasma metabolic signature (Patient PGB_26), Figure S34: Network-based patient specific plasma metabolic signature (Patient PGB_27), Figure S35: Network-based patient specific plasma metabolic signature (Patient PGB_28), Figure S36: Network-based patient specific plasma metabolic signature (Patient PGB_29), Table S1: Metabolites List used in Targeted Metabolomics, Table S2: Sources and IMS parameters for Ion Mobility Mass Spectrometry analyses, Table S3: Cross Collision Section Calculation Curve, Table S4: Variable metadata (Untargeted LC-IMS-MS Metabolomics), Table S5: Variable metadata (Untargeted FT-ICR-MS Metabolomics), Table S6: Data matrix (Targeted LC-MS/MS Metabolomics), Table S7: Data matrix (Untargeted LC-IMS-MS Metabolomics), Table S8: Data matrix (Untargeted FT-ICR-MS Metabolomics), Table S9: Cohorte Overview, Table S10: Sample Spearman correlation (Targeted LC-MS/MS Metabolomics), Table S11: Sample Spearman correlation (Untargeted LC-IMS-MS Metabolomics, Table S12: Sample Spearman correlation (Untargeted FT-ICR-MS Metabolomics), Table S13: Principal Component Analysis Scores, Table S14: Differential Analysis Results, Table S15: Analysis of overlap with litterature, Table S16: Spearman Metabolite Correlation Matrix, Table S17: Spearman Correlations with circulating free DNA, Table S18: Partial Correlation Matrix including all samples, Table S19: Pruned netwrok using all samples network, Table S20: Partial Correlation Matrix including only control samples, Table S21: Patient specfic pruned networks, Table S22: Consensus network, Table S23: All Random Forest Models.
