# Supplementary material for: Integrative Metabolomics Reveals Deep Tissue and Systemic Metabolic Remodeling in Glioblastoma
Source: Cancers (Basel). 2021 Oct 14;13(20):5157. doi: 10.3390/cancers13205157 (PMC8534284; doi:10.3390/cancers13205157)
Supplement: Supplementary file 1 [file cancers-13-05157-s001.zip › cancers-1394141 supplementary figures after proof.pdf]

Supplementary material: Integrative Metabolomics Reveals Deep Tissue and Systemic Metabolic Remodeling in Glioblastoma

Vianney Gilard, Justine Ferey, Florent Marguet, Maxime Fontanilles, Franklin Ducatez, Carine Pilon, Céline Lesueur, Tony Pereira, Carole Basset, Isabelle Schmitz-Afonso, Frédéric Di Fioré, Annie Laquerrière, Carlos Afonso, Stéphane Derrey, Stéphane Marret, Soumeya Bekri and Abdellah Tebani

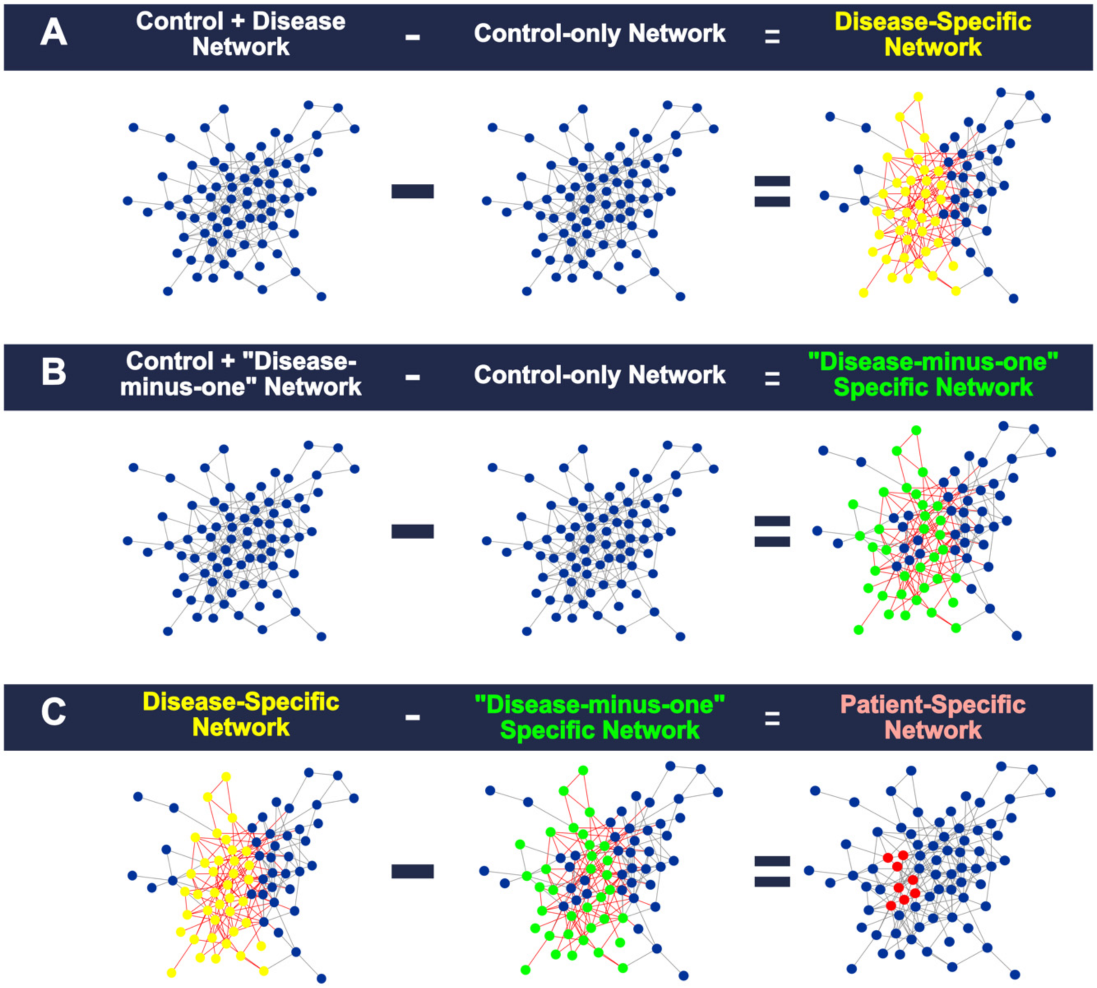

Figure S1. Network analysis overview. Networks are built using Partial Correlation. The first network (“disease-control” network) is learned from disease profiles and control profiles. A second network composed of only control (“control-only” network). A third network is composed of control profiles and disease excluding one patient at a time (“Disease-minus-one” network). This third network is built iteratively to cover all the patients. A final pruning stage subtracts edges from the disease-control network that are also found either in A) the control-only network, outputting the disease-specific network B) “Disease-minus-one” specific network or C) Patient-specific network.

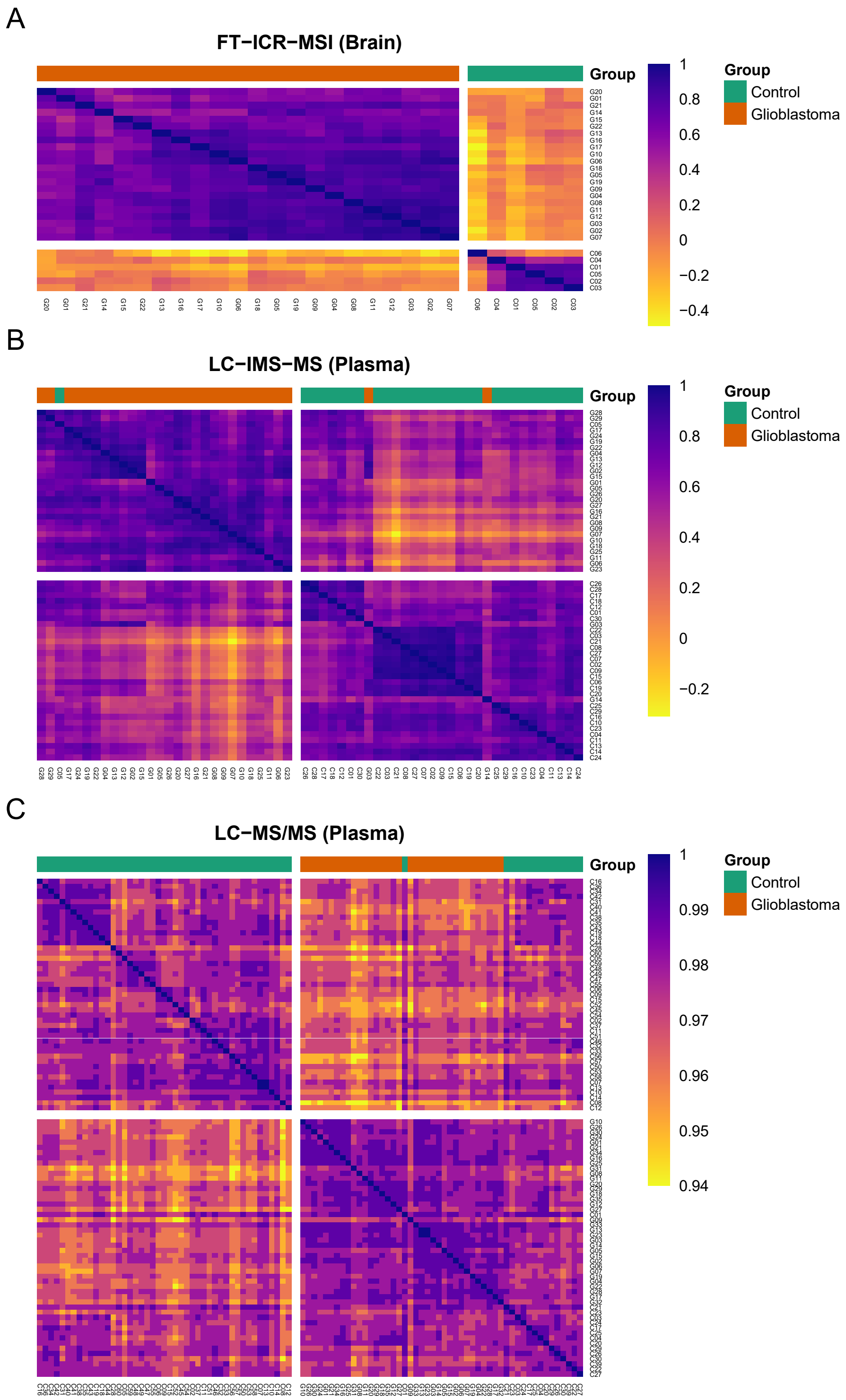

Figure S2. Spearman correlations between samples.

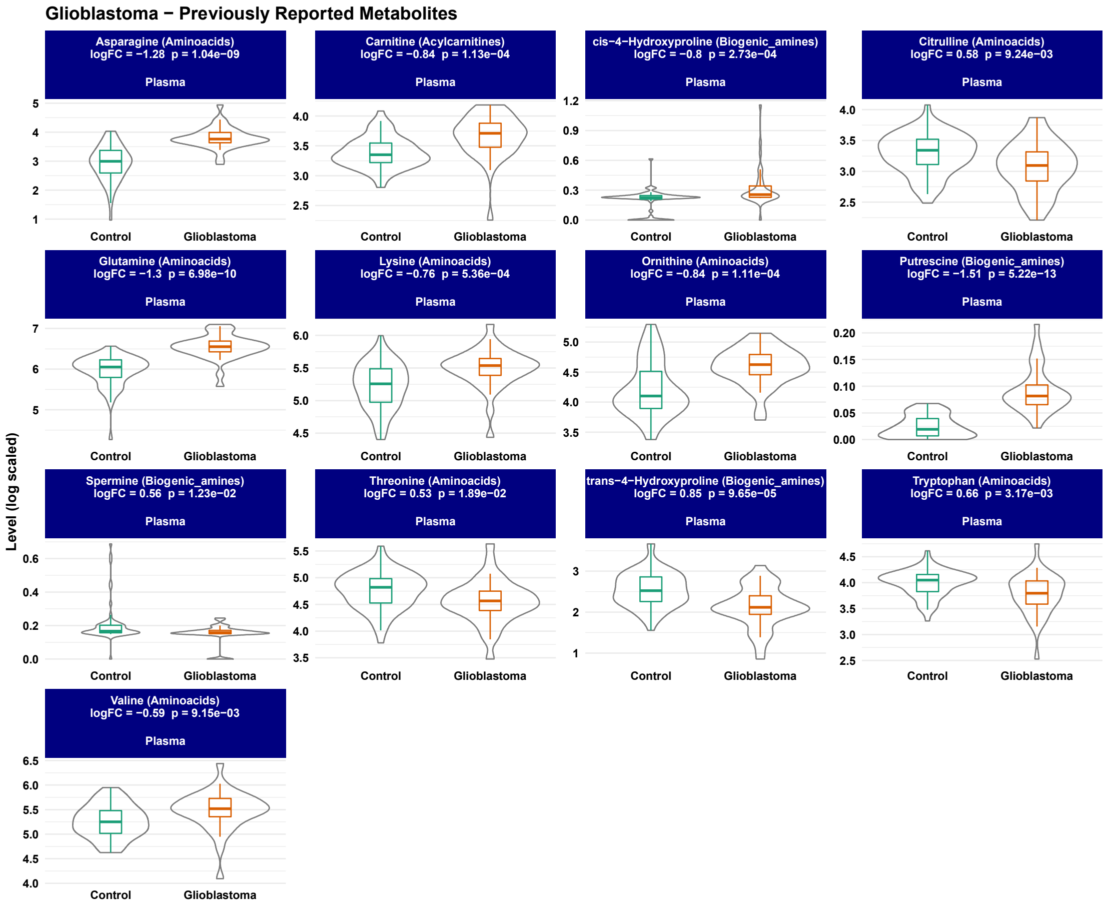

Figure S3. Boxplots of previously reported metabolites.

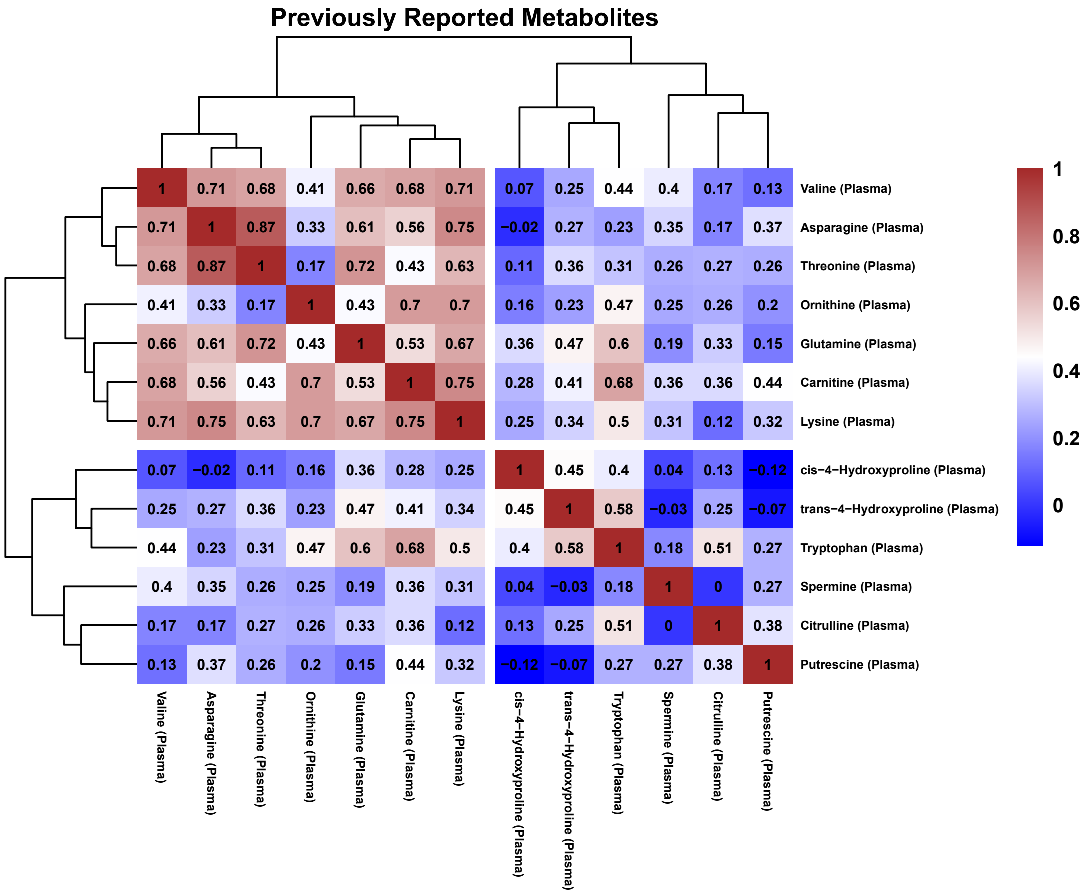

Figure S4. Heatmap of Spearman correlations of previously reported metabolites.

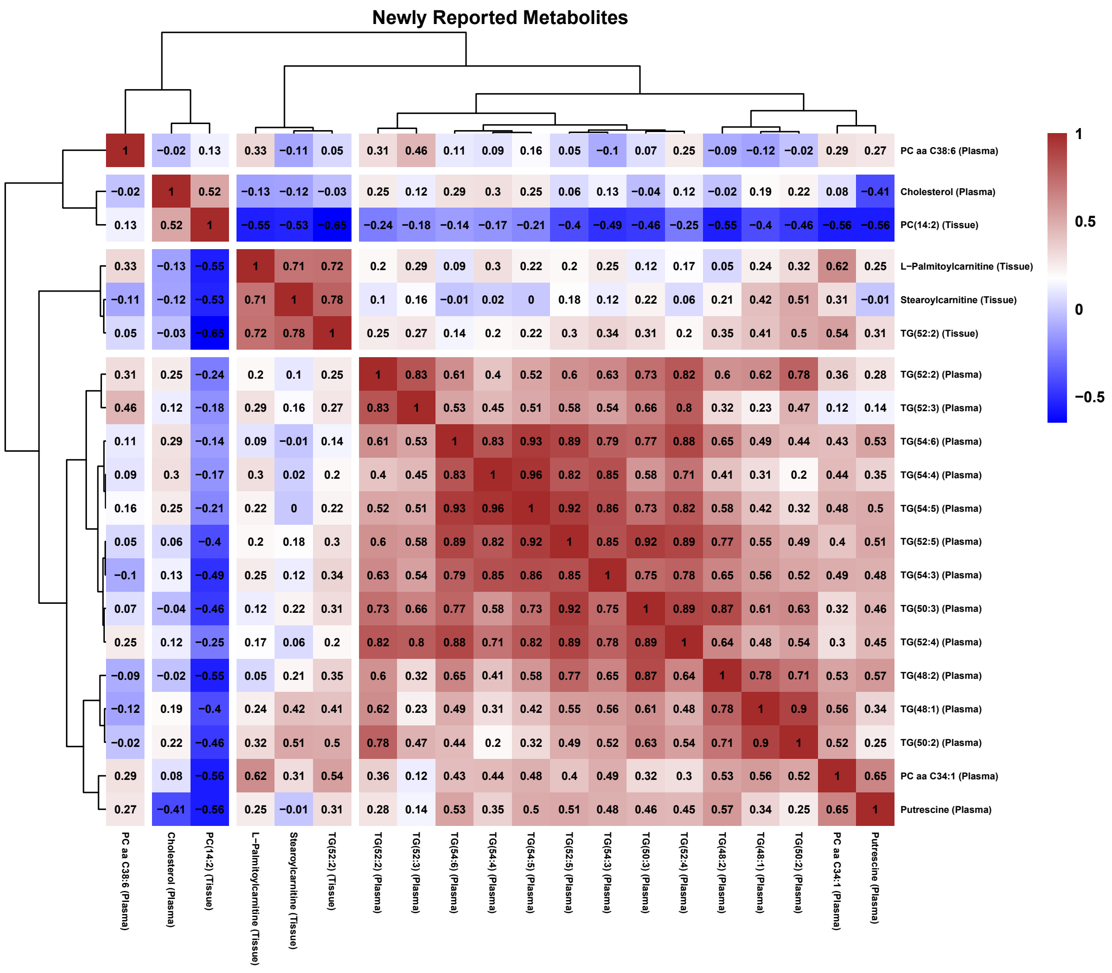

Figure S5. Heatmap of Spearman correlations of newly reported metabolites.

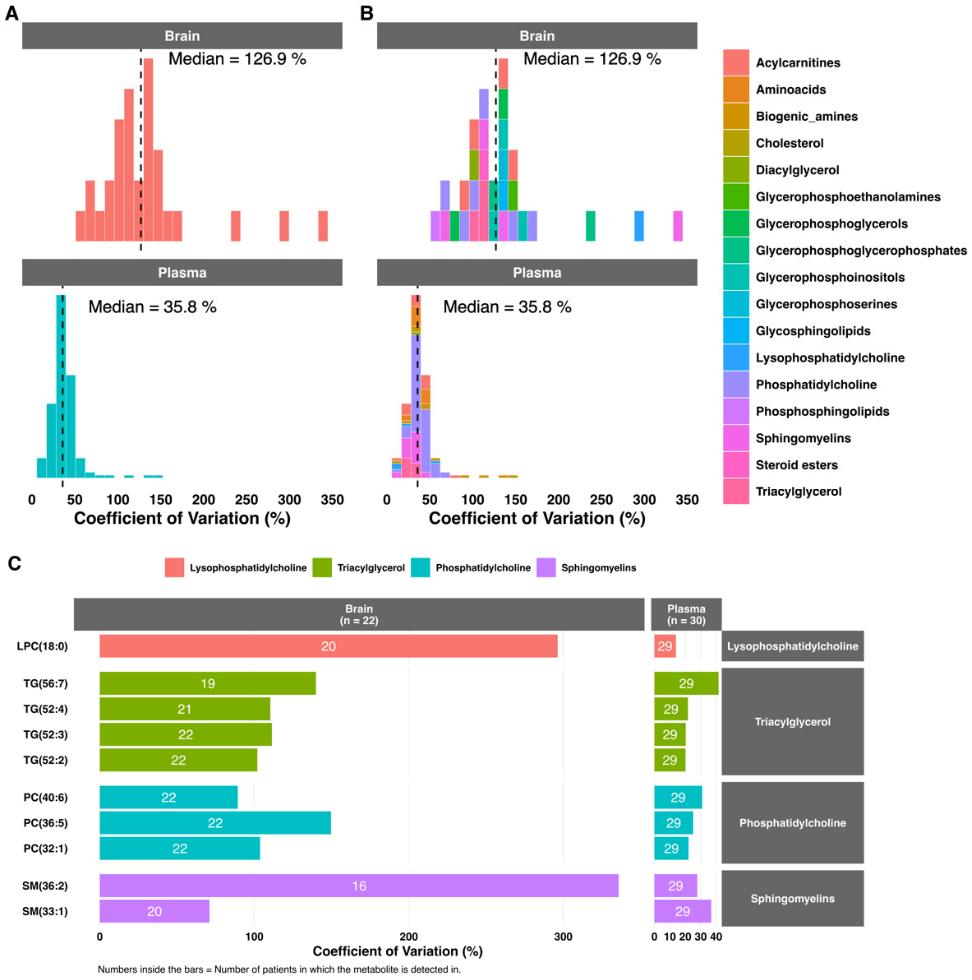

Figure S6. Intra-group (GBM) variation. A) Histogram distribution of coefficient of variation in brain and plasma (Colored according to matrice). B) Histogram distribution of coefficient of variation in brain and plasma (Colored according to biochemical classes). C) Coefficient of variation of the 10 lipids correlated between plasma and brain samples.

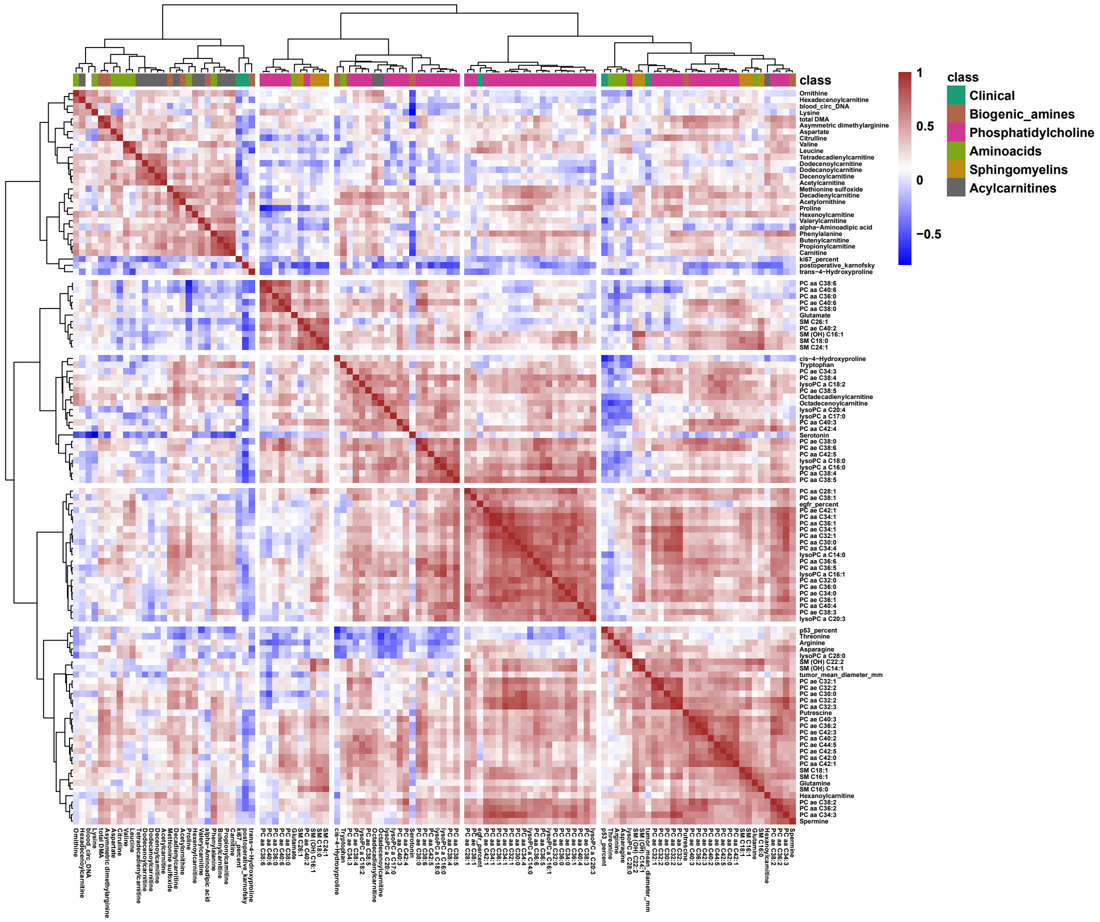

Figure S7. Heatmap of Spearman correlations between all significant metabolites.

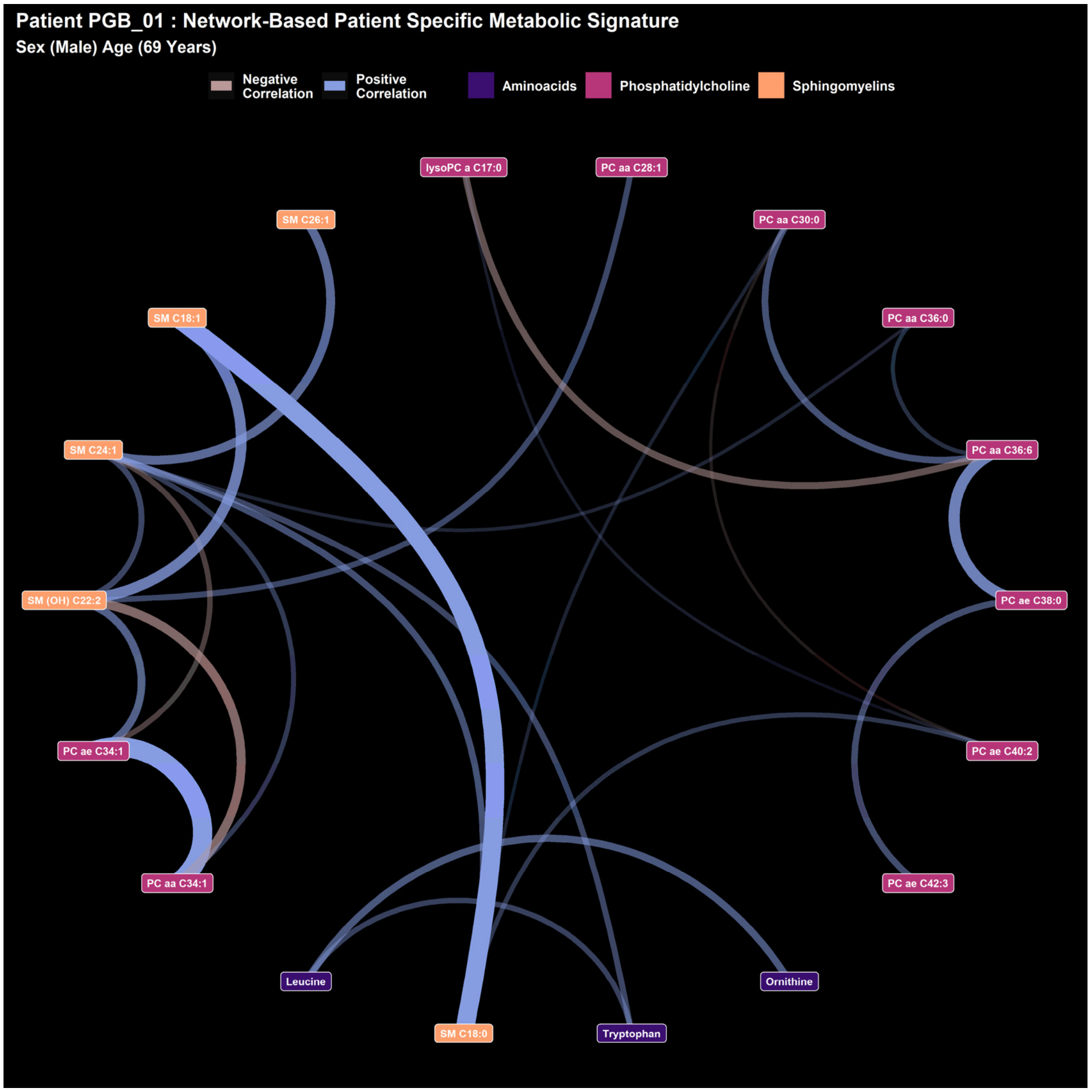

Figure S8. Network-based patient specific plasma metabolic signature (Patient PGB_01). The signature includes 18 metabolites; 10 phosphatidylcholines, 5 sphingomyelin and 3 aminoacids.

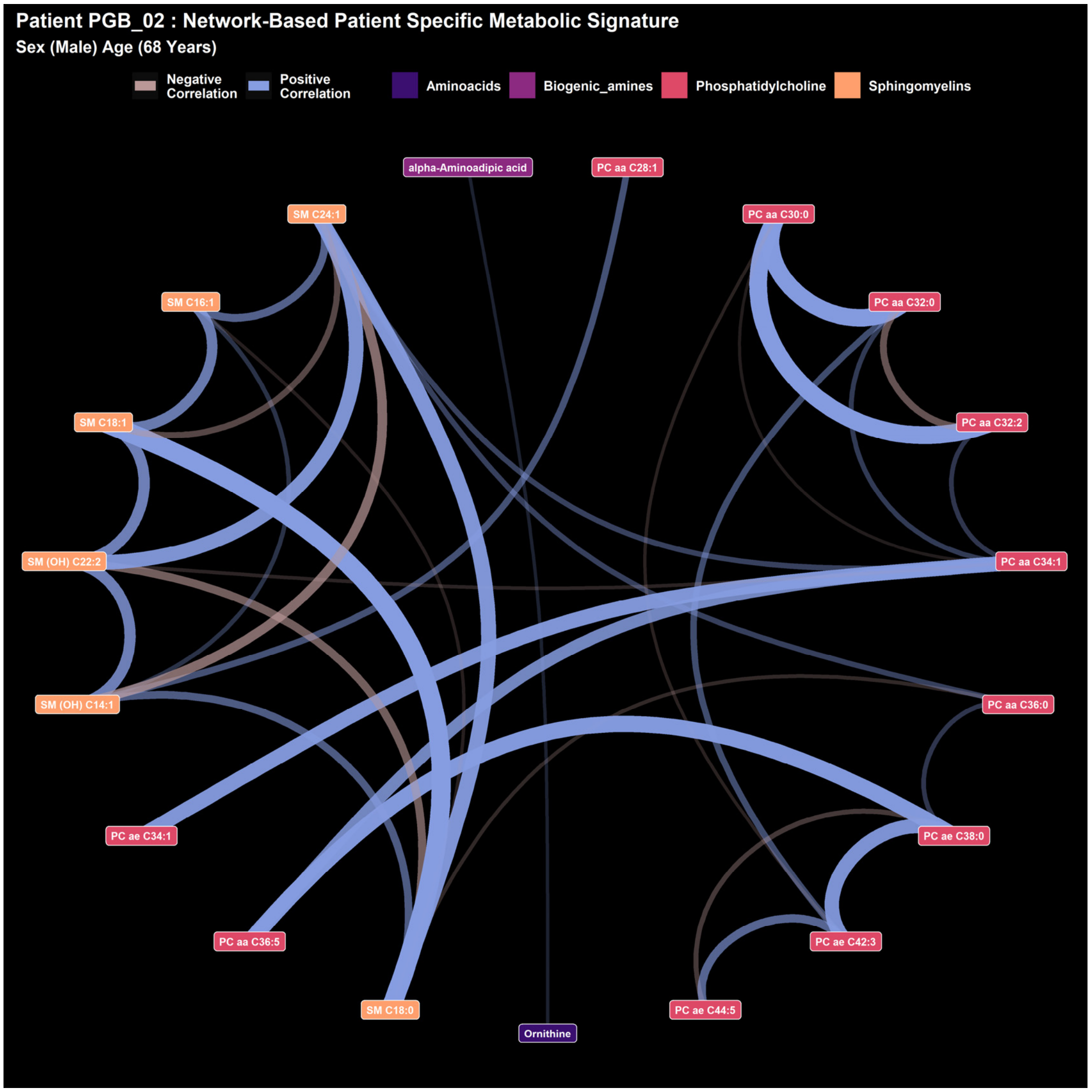

Figure S9. Network-based patient specific plasma metabolic signature (Patient PGB_02). The signature includes 19 metabolites; 11 phosphatidylcholines, 6 sphingomyelin, 1 aminoacid and 1 biogenic amine.

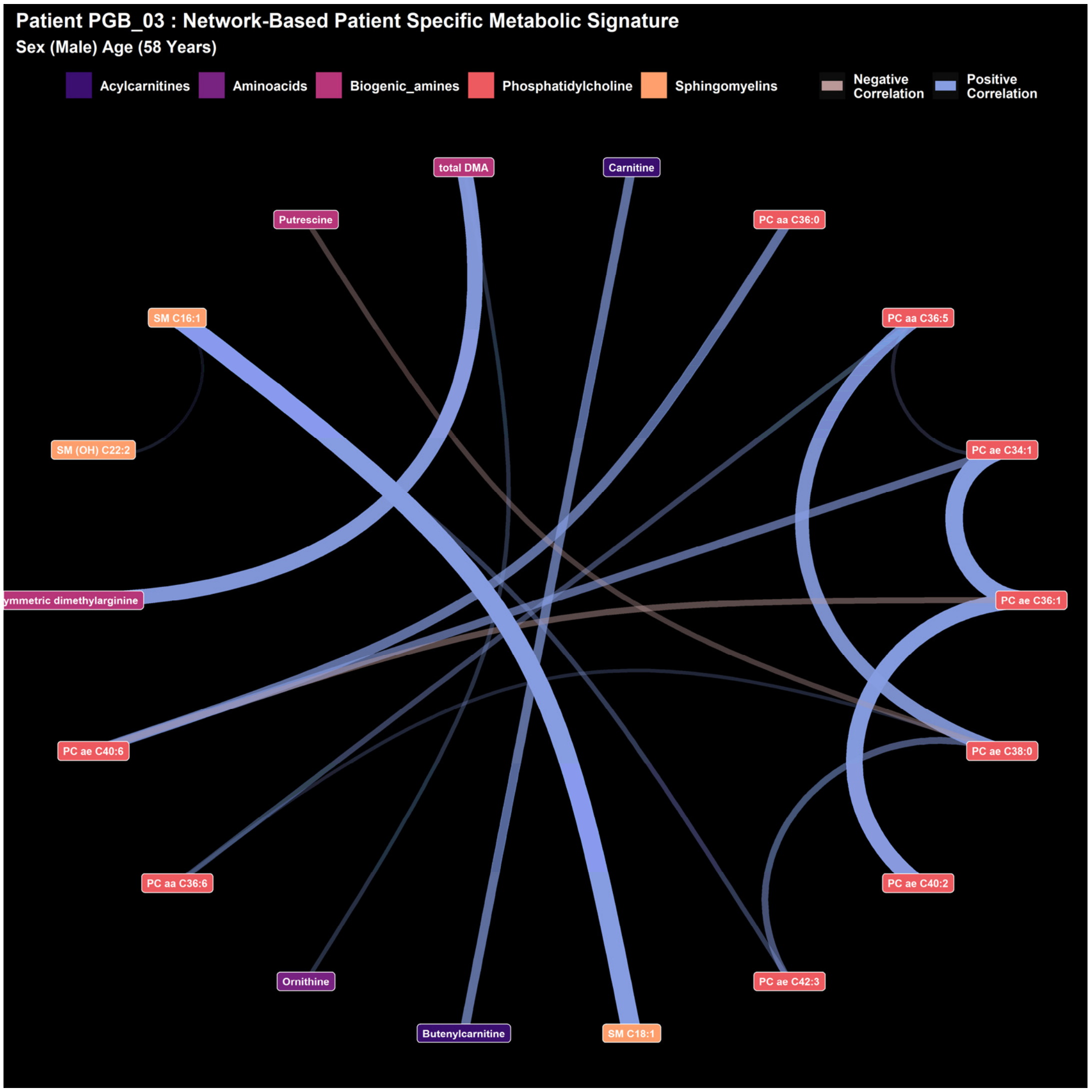

Figure S10. Network-based patient specific plasma metabolic signature (Patient PGB_03). The signature includes 18 metabolites; 9 phosphatidylcholines, 3 sphingomyelin, 1 aminoacid and 3 biogenic amines.

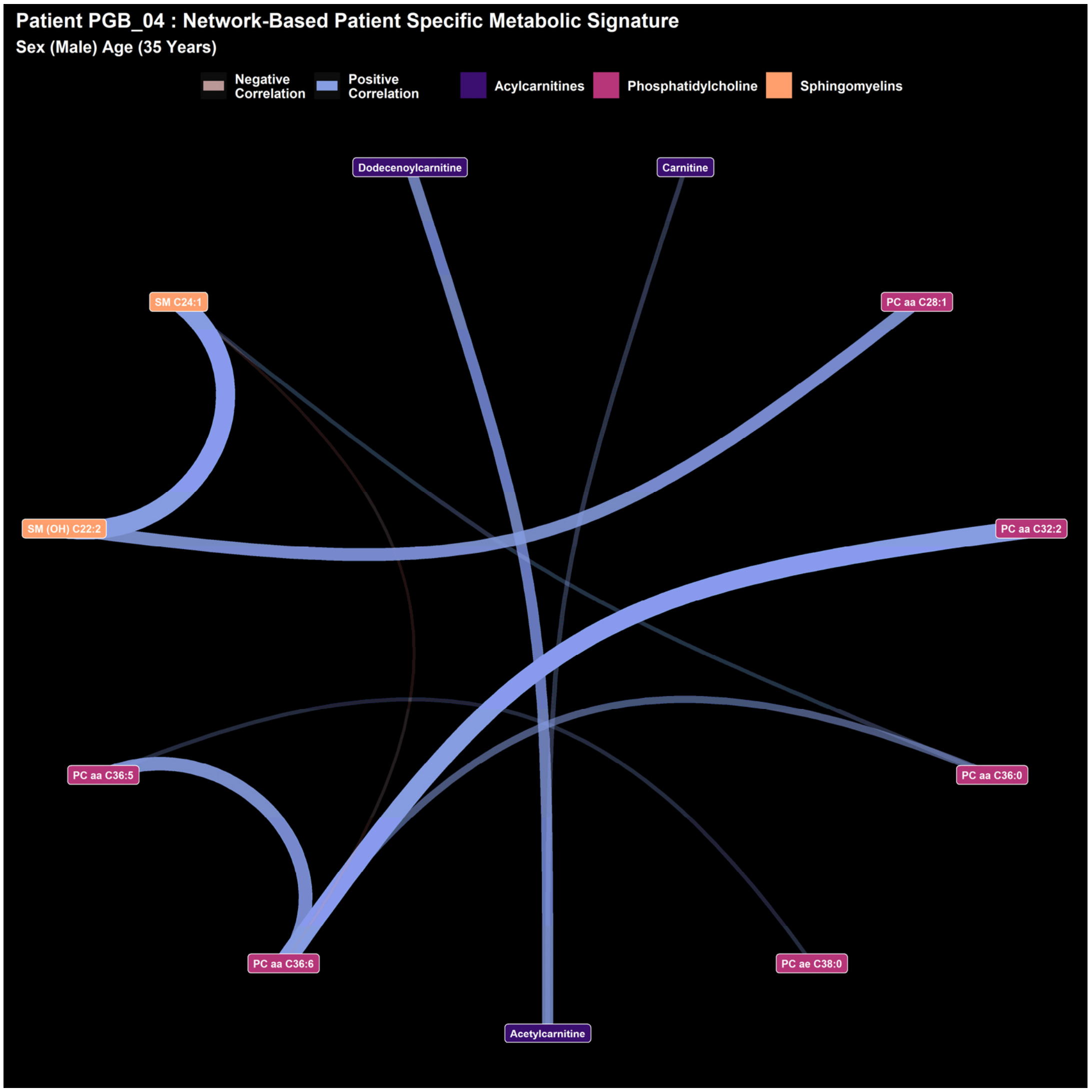

Figure S11. Network-based patient specific plasma metabolic signature (Patient PGB_04). The signature includes 11 metabolites; 6 phosphatidylcholines, 3 acylcarnitines and 2 sphingomyelins.

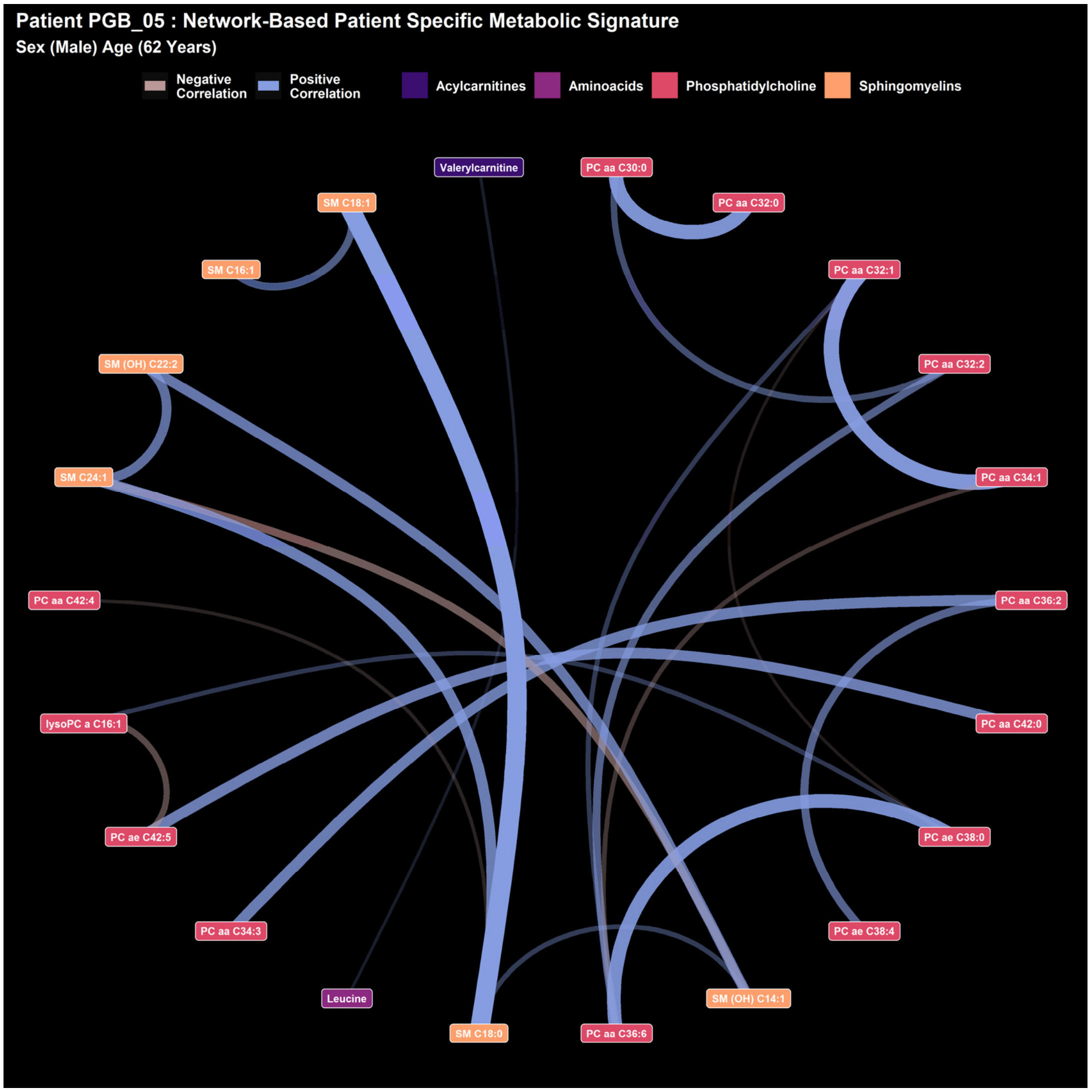

Figure S12. Network-based patient specific plasma metabolic signature (Patient PGB_05). The signature includes 22 metabolites; 14 phosphatidylcholines, 6 sphingomyelin, 1 aminoacid and 1 acylcarnitine.

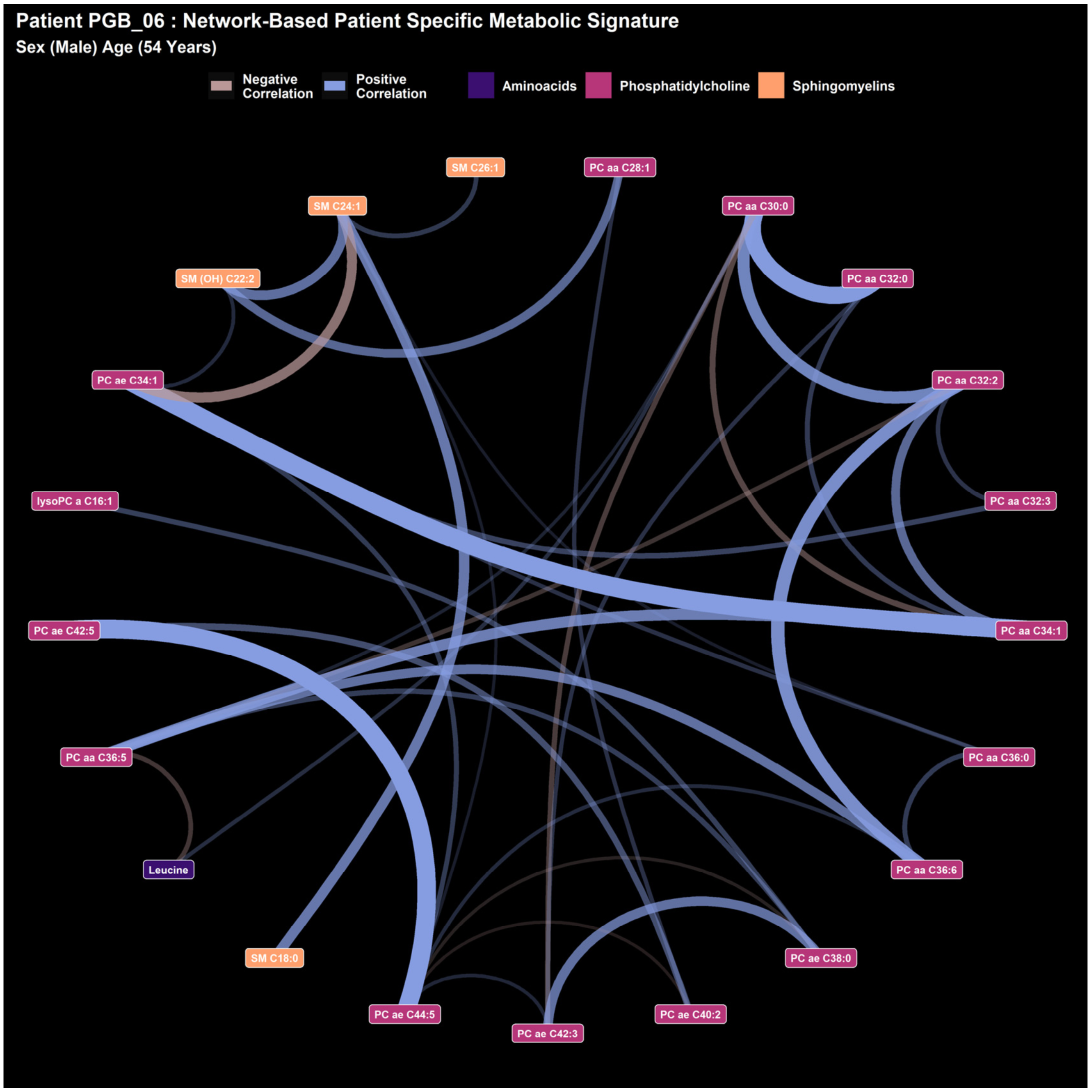

Figure S13. Network-based patient specific plasma metabolic signature (Patient PGB_06). The signature includes 21 metabolites; 16 phosphatidylcholines, 4 sphingomyelins and 1 aminoacid.

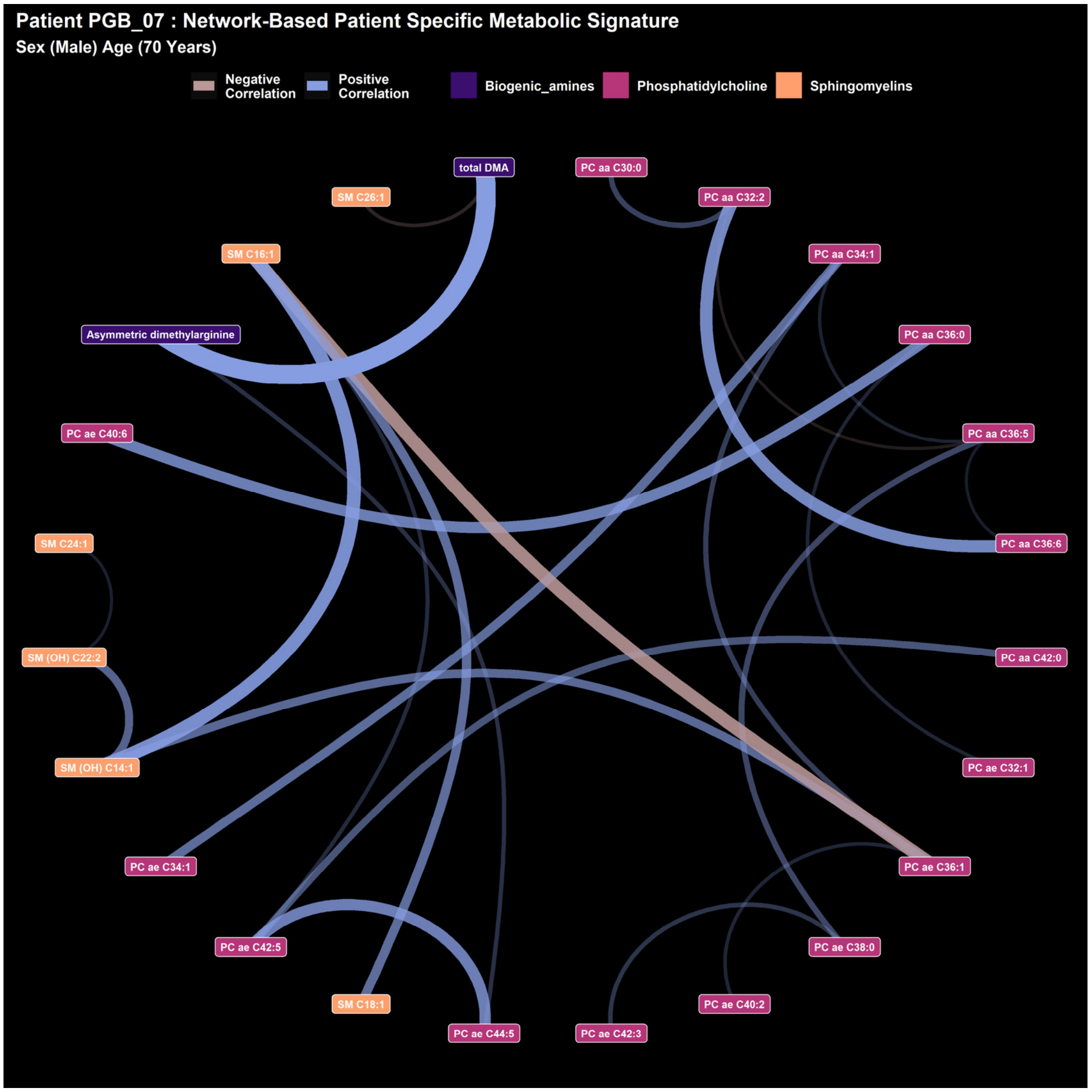

Figure S14. Network-based patient specific plasma metabolic signature (Patient PGB_07). The signature includes 24 metabolites; 16 phosphatidylcholines, 6 sphingomyelins and 2 biogenic amines.

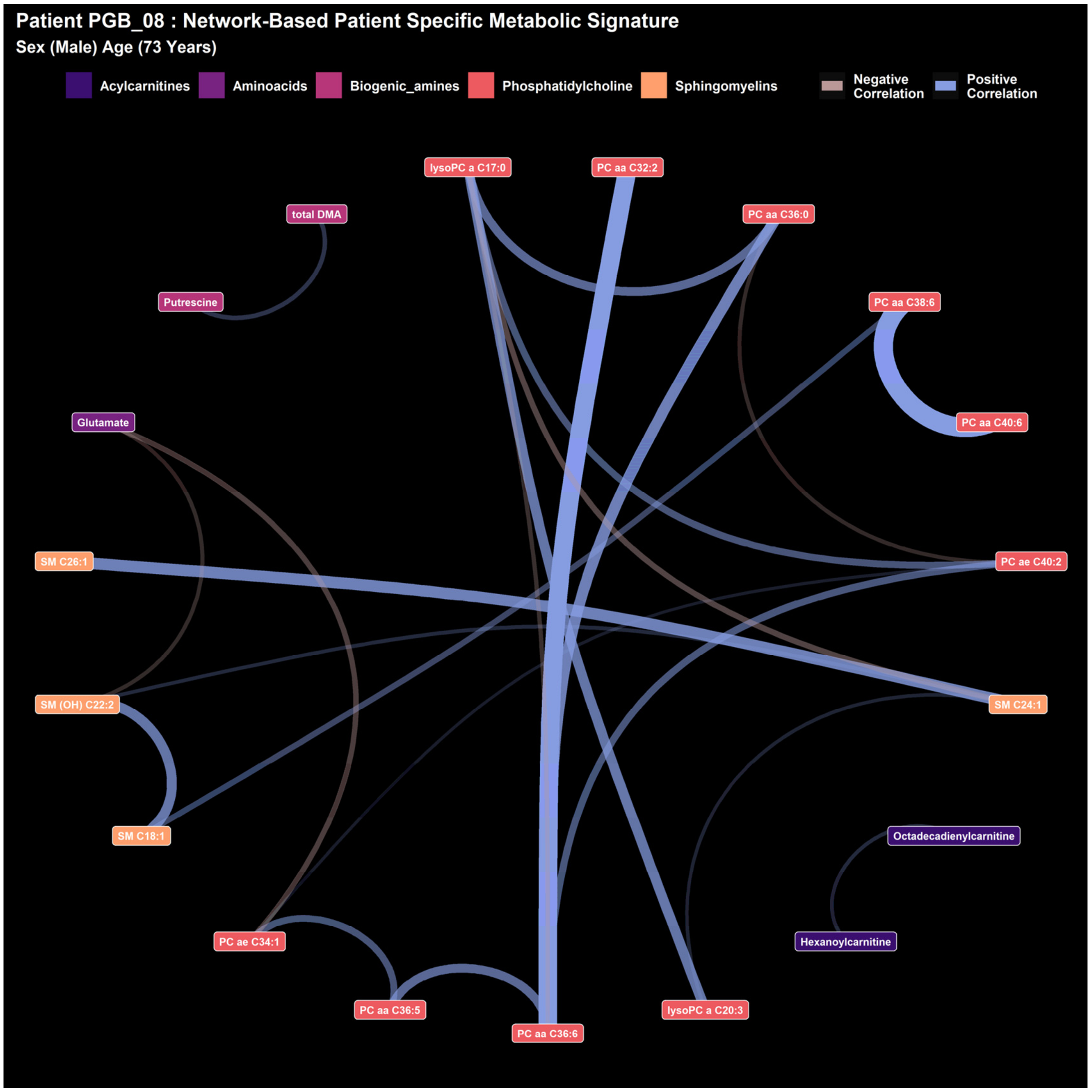

Figure S15. Network-based patient specific plasma metabolic signature (Patient PGB_08). The signature includes 19 metabolites; 10 phosphatidylcholines, 4 sphingomyelins, 2 acylcarnitines, 2 biogenic amines and 1 aminoacid.

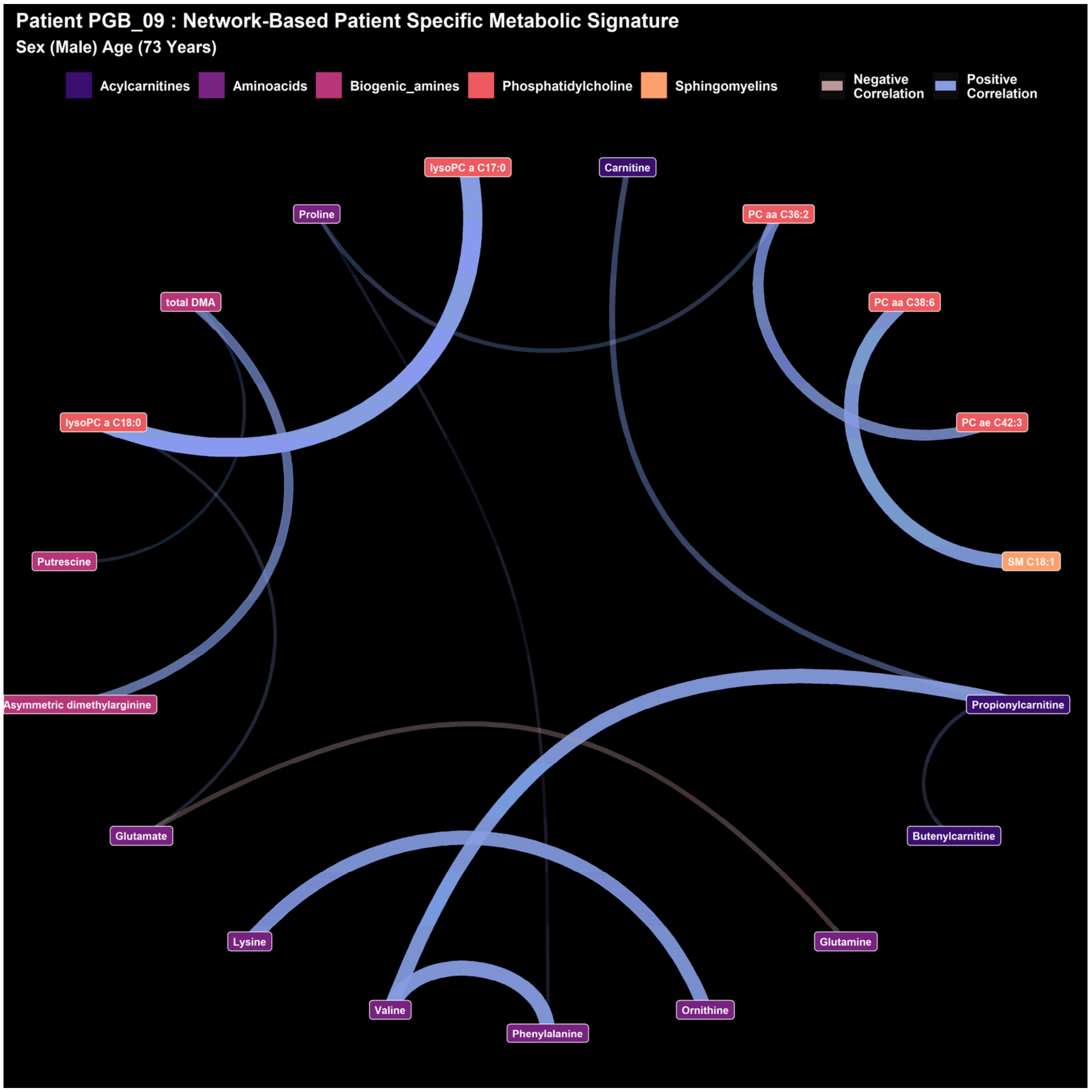

Figure S16. Network-based patient specific plasma metabolic signature (Patient PGB_09). The signature includes 19 metabolites; 5 phosphatidylcholines, 7 aminoacids, 3 acylcarnitines, 3 biogenic amines and 1 sphingomyelin.

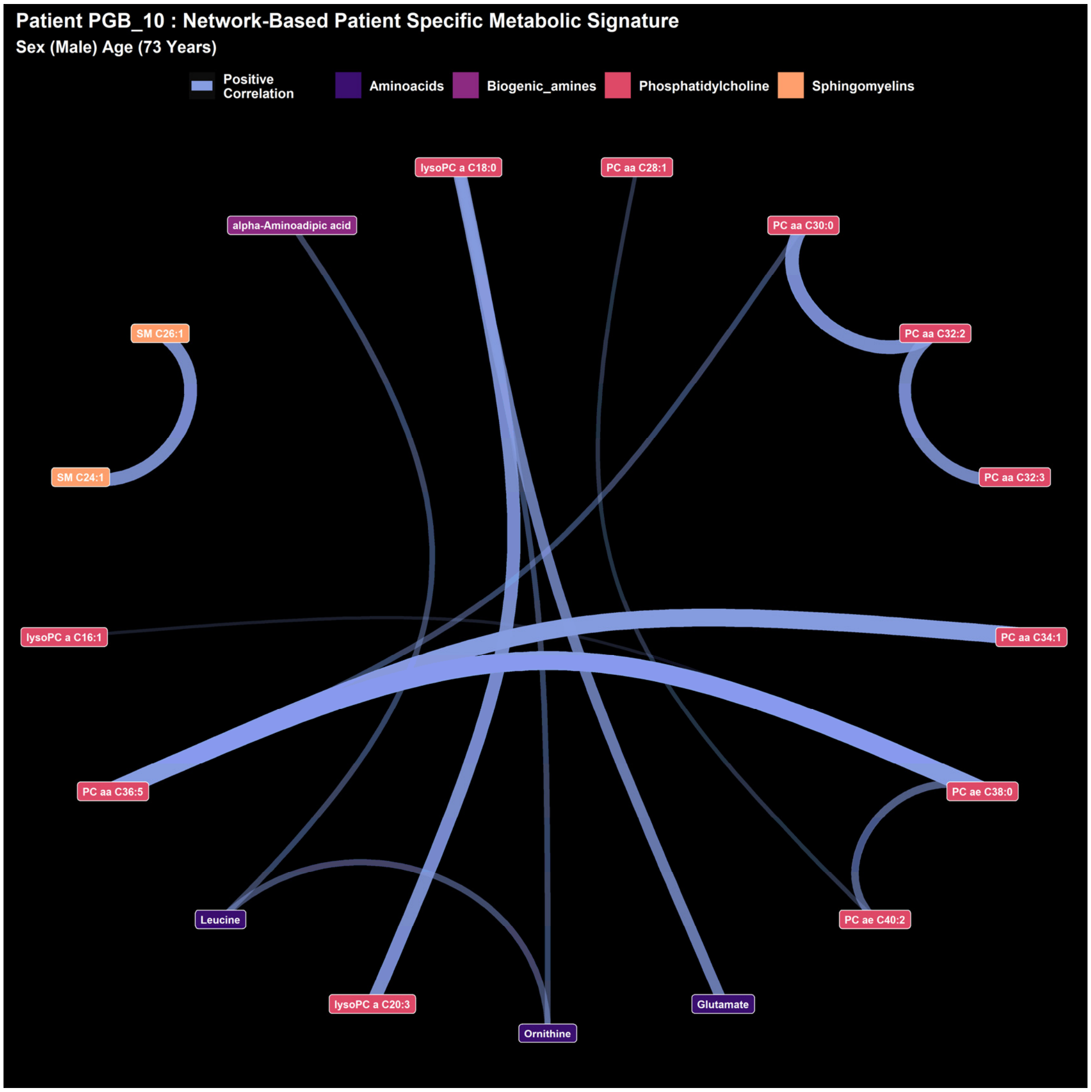

Figure S17. Network-based patient specific plasma metabolic signature (Patient PGB_10). The signature includes 17 metabolites; 11 phosphatidylcholines, 3 aminoacids, 1 biogenic amine and 2 sphingomyelins.

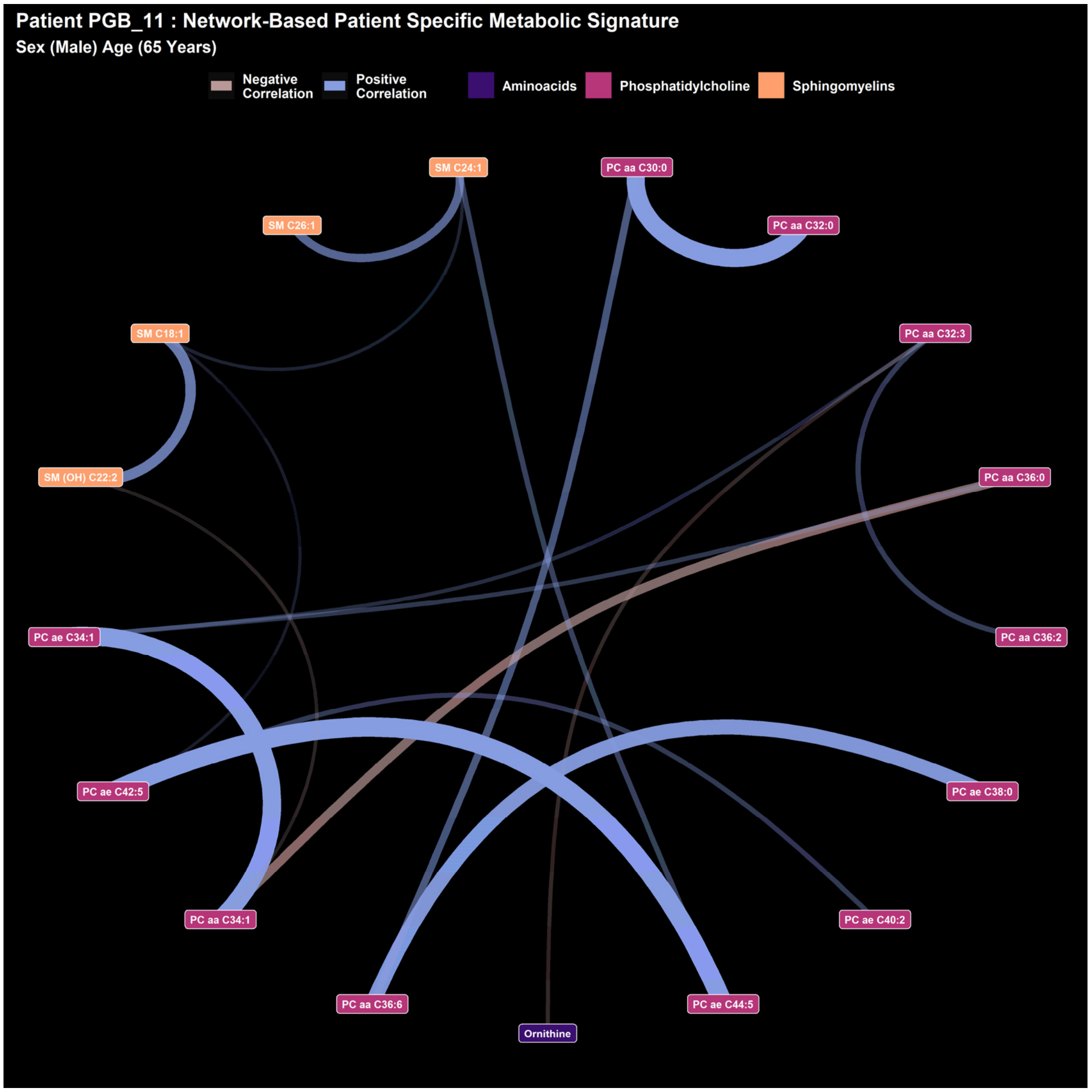

Figure S18. Network-based patient specific plasma metabolic signature (Patient PGB_11). The signature includes 17 metabolites; 12 phosphatidylcholines, 1 aminoacid, and 4 sphingomyelins.

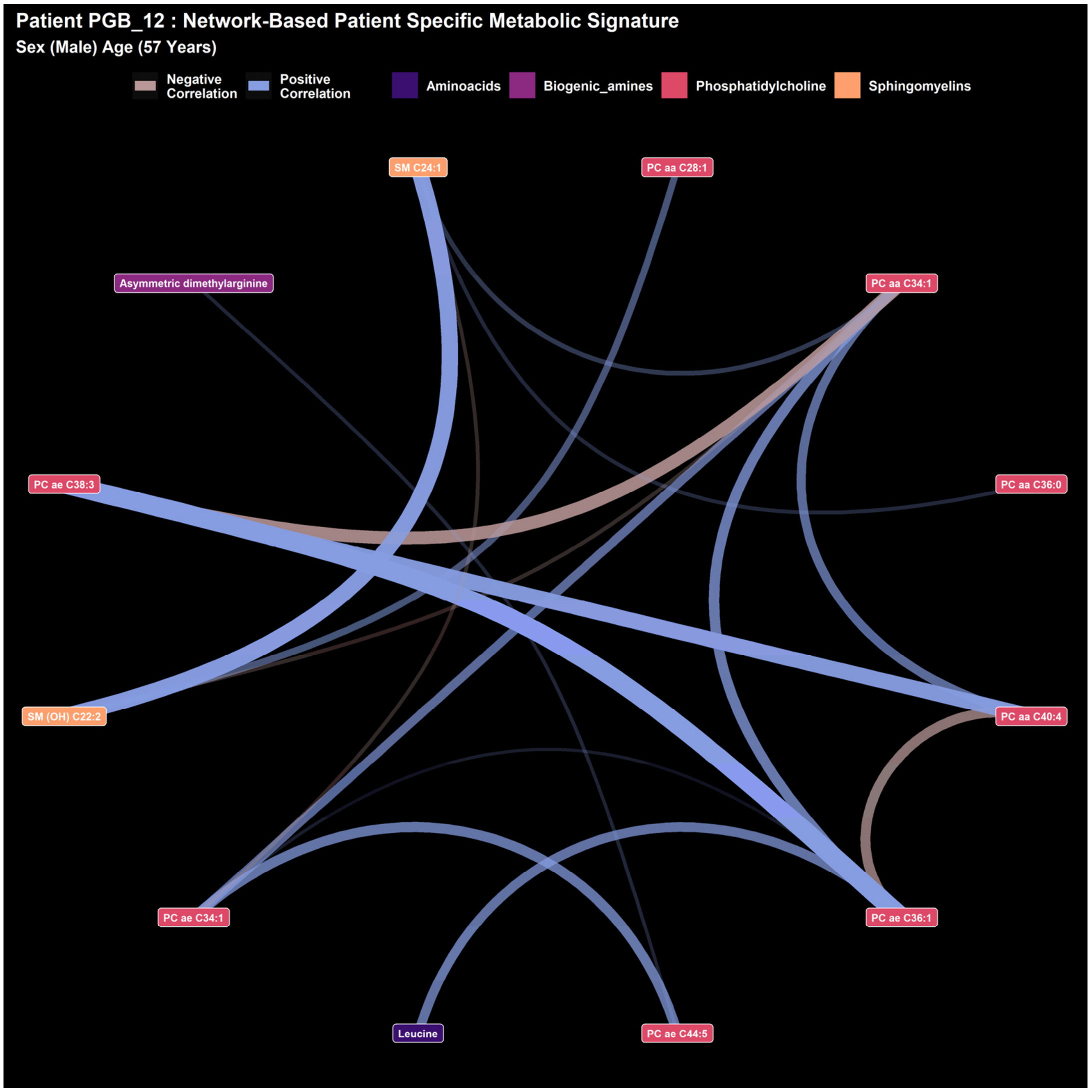

Figure S19. Network-based patient specific plasma metabolic signature (Patient PGB_12). The signature includes 12 metabolites; 8 phosphatidylcholines, 2 aminoacids, 1 biogenic amine and 2 sphingomyelins.

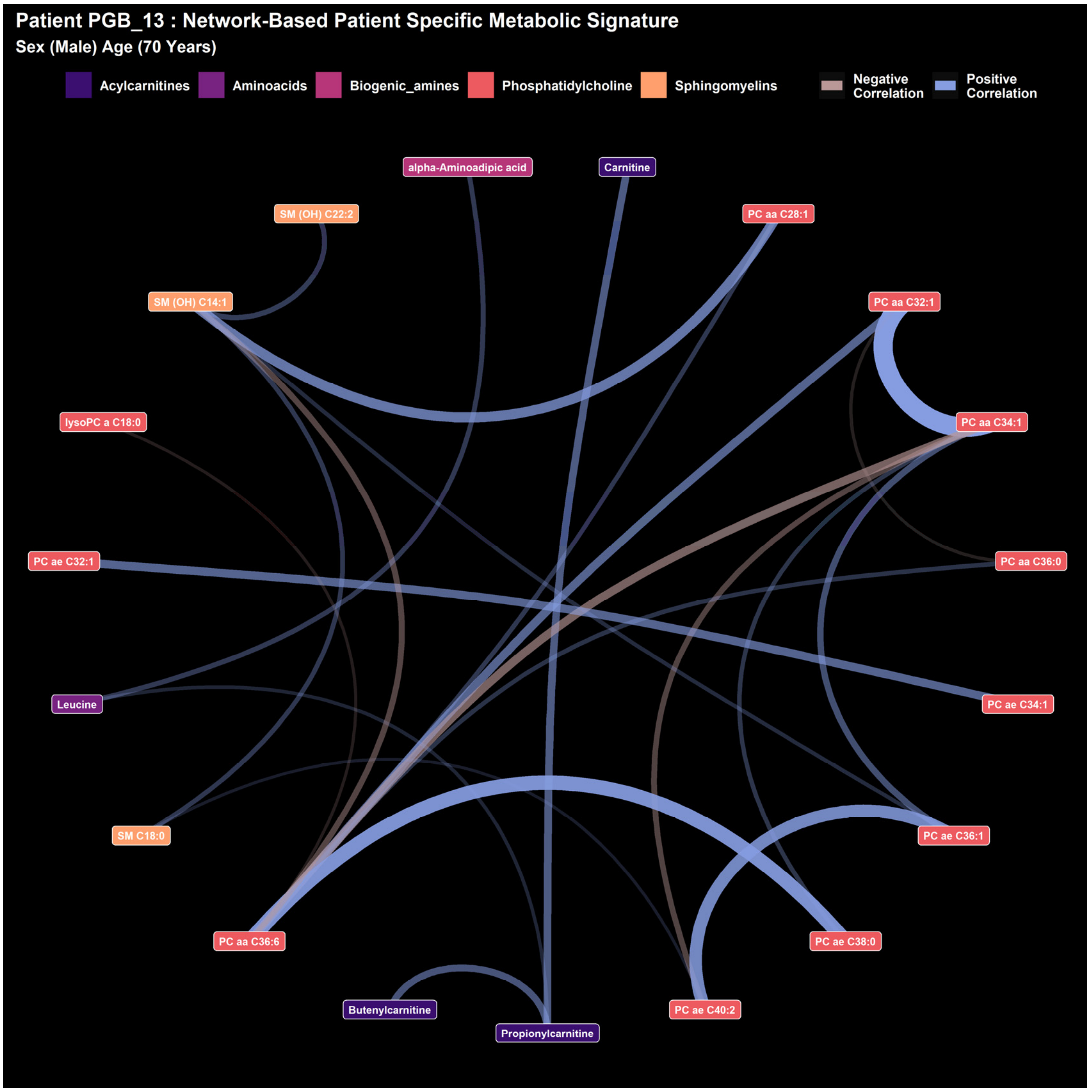

Figure S20. Network-based patient specific plasma metabolic signature (Patient PGB_13). The signature includes 19 metabolites; 11 phosphatidylcholines, 3 aminoacids, 3 acylcarnitines, 1 biogenic amine and 3 sphingomyelins.

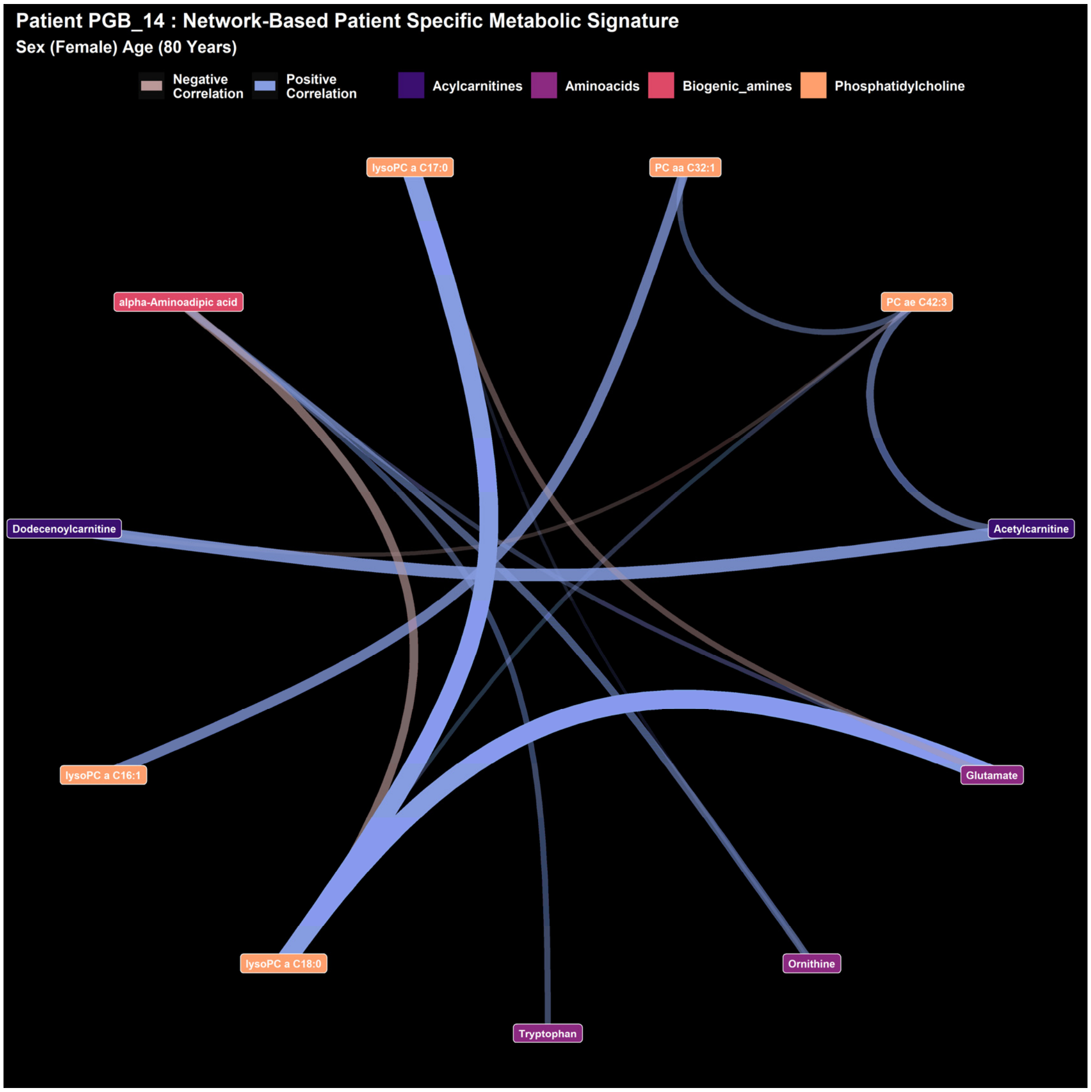

Figure S21. Network-based patient specific plasma metabolic signature (Patient PGB_14). The signature includes 11 metabolites; 5 phosphatidylcholines, 3 aminoacids, 2 acylcarnitines and 1 biogenic amine.

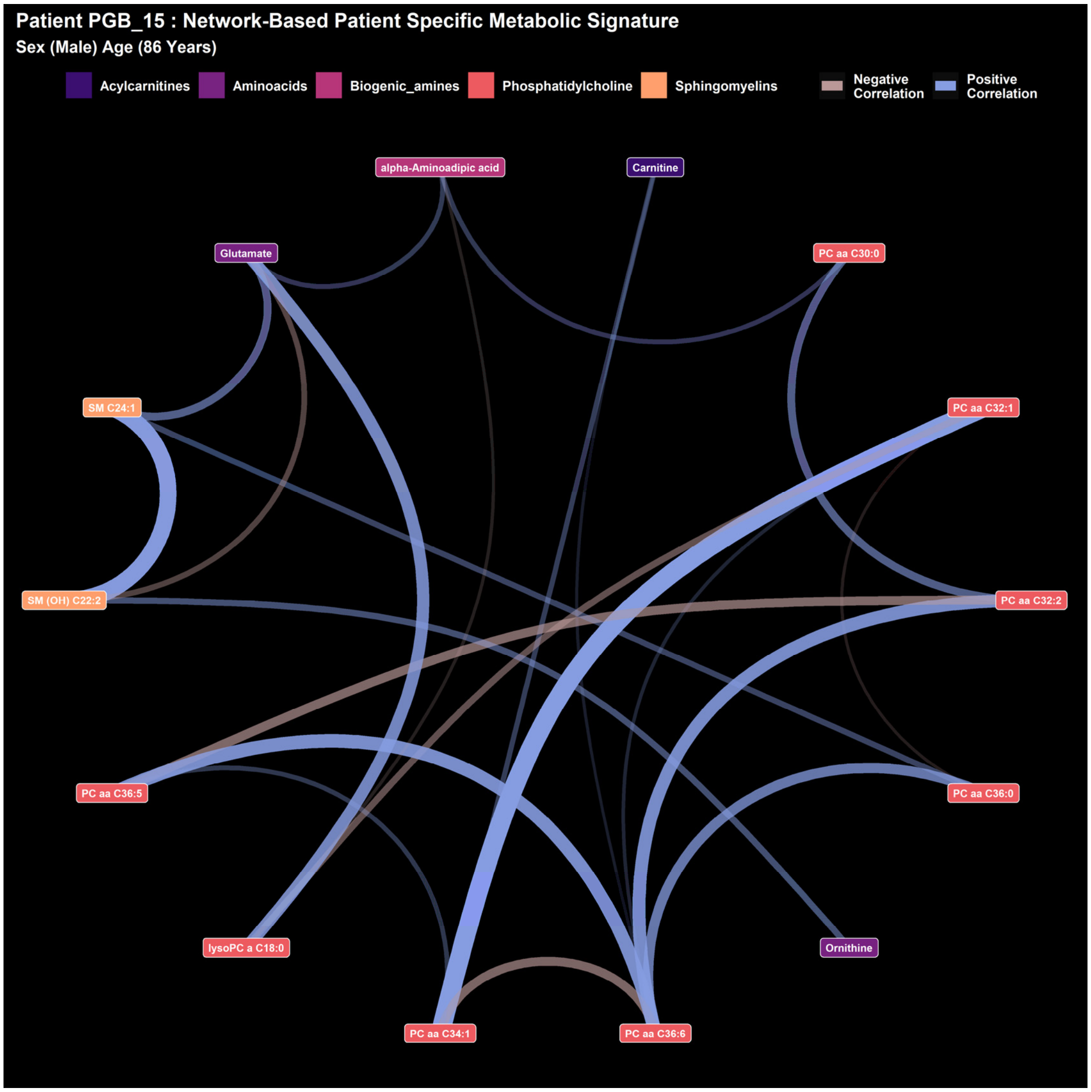

Figure S22. Network-based patient specific plasma metabolic signature (Patient PGB_15). The signature includes 14 metabolites; 8 phosphatidylcholines, 2 aminoacids, 1 acylcarnitine, 1 biogenic amine and 2 sphingomyelins.

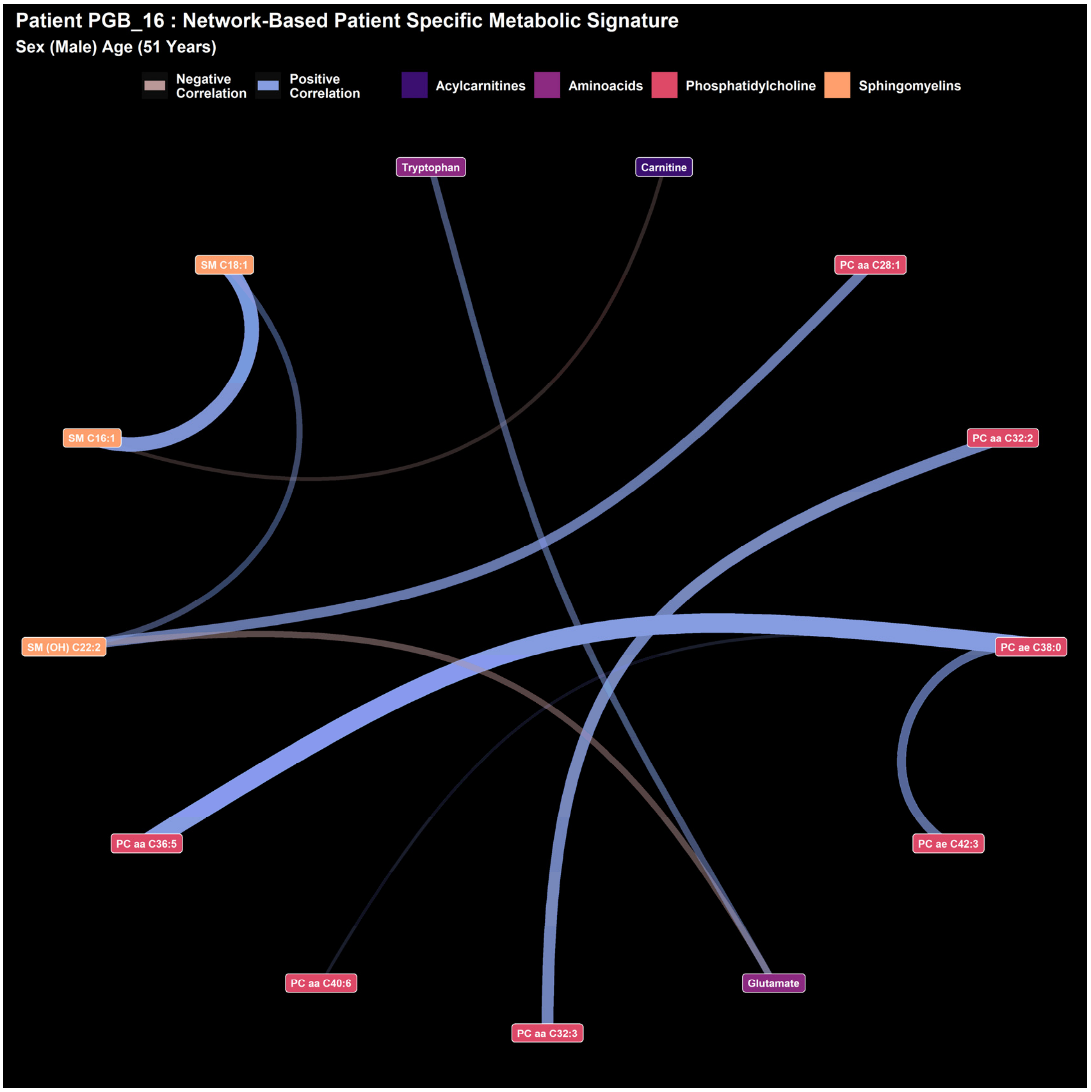

Figure S23. Network-based patient specific plasma metabolic signature (Patient PGB_16). The signature includes 13 metabolites; 7 phosphatidylcholines, 2 aminoacids, 1 acylcarnitine and 3 sphingomyelins.

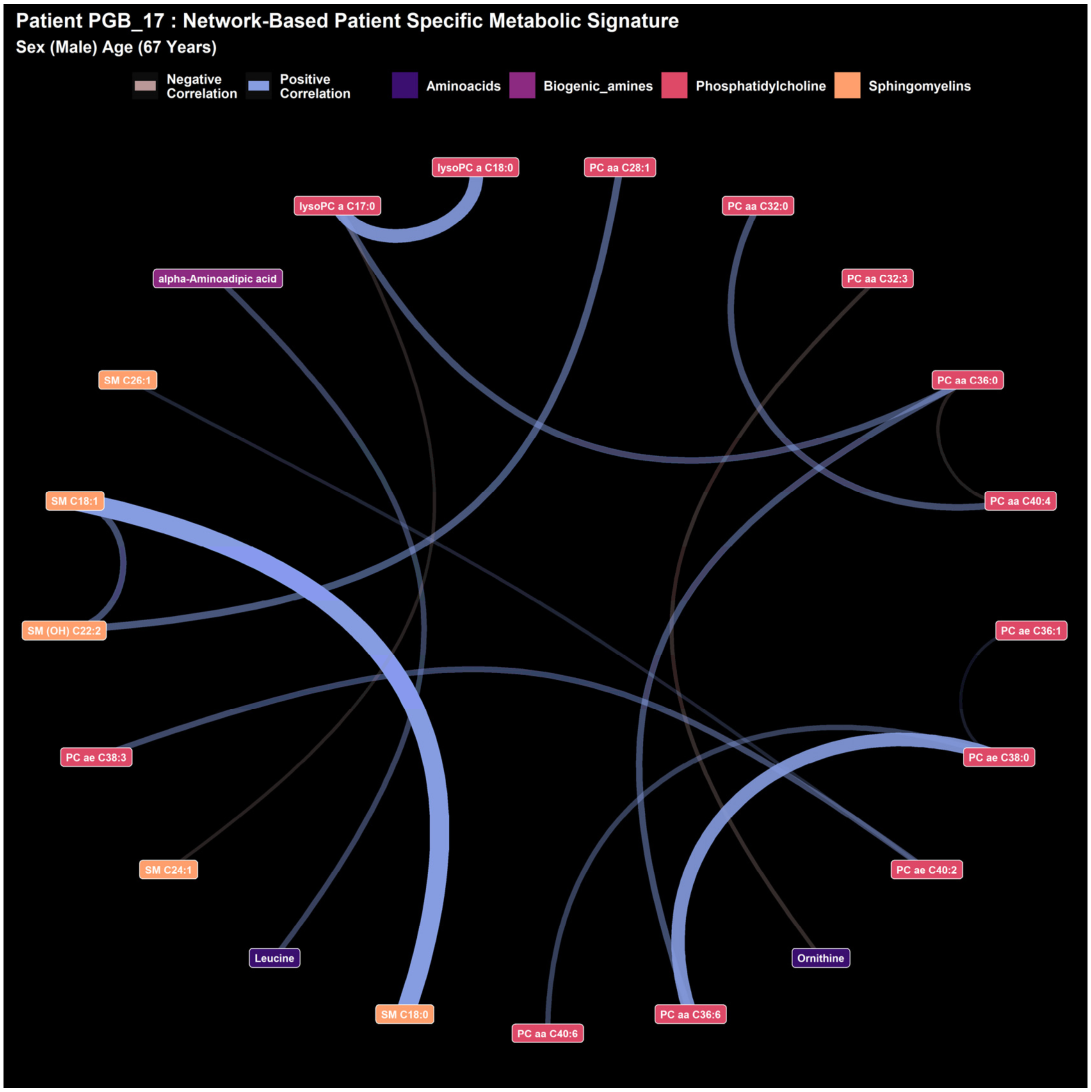

Figure S24. Network-based patient specific plasma metabolic signature (Patient PGB_17). The signature includes 21 metabolites; 13 phosphatidylcholines, 2 aminoacids, 1 biogenic amine and 5 sphingomyelins.

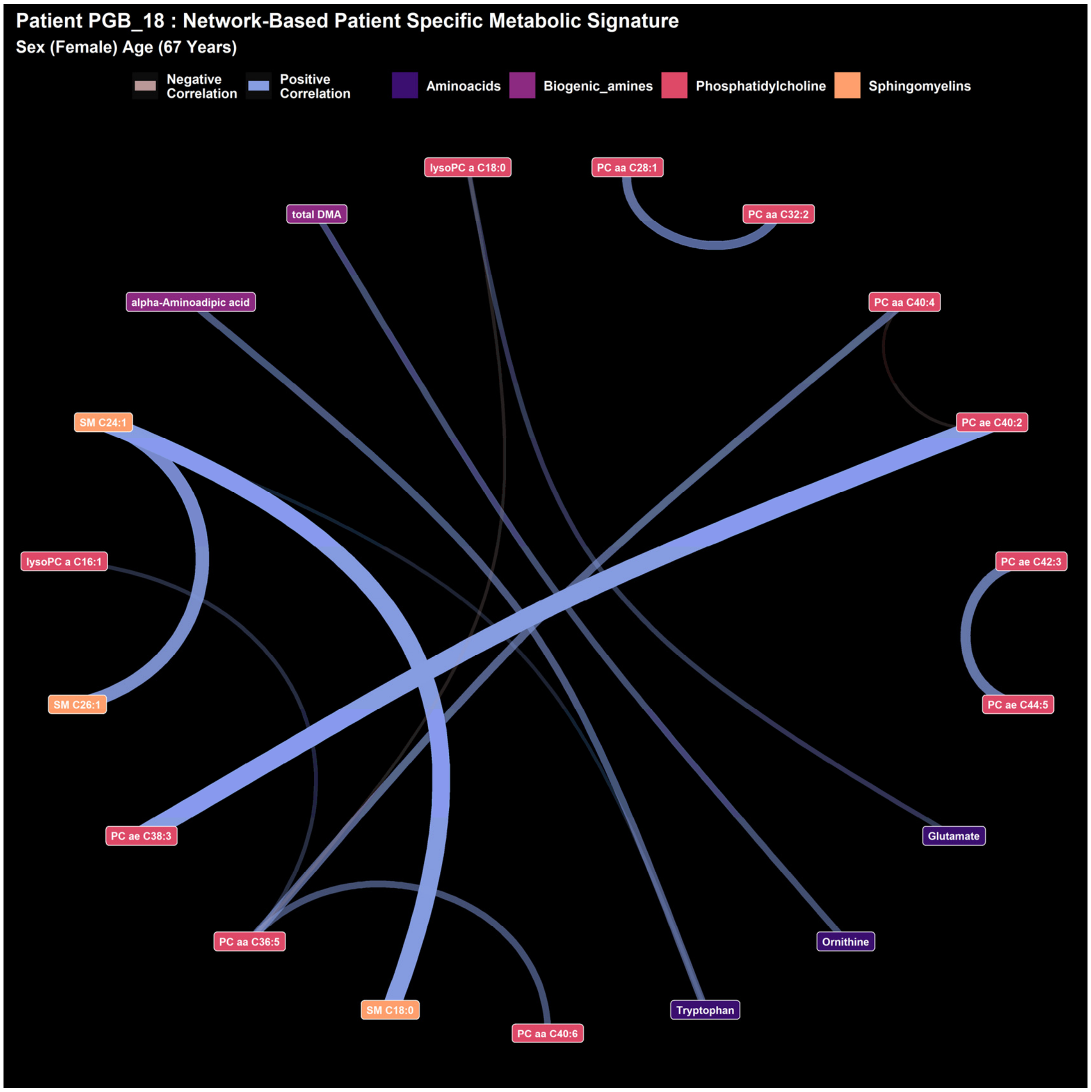

Figure S25. Network-based patient specific plasma metabolic signature (Patient PGB_18). The signature includes 19 metabolites; 11 phosphatidylcholines, 3 aminoacids, 2 biogenic amines and 3 sphingomyelins.

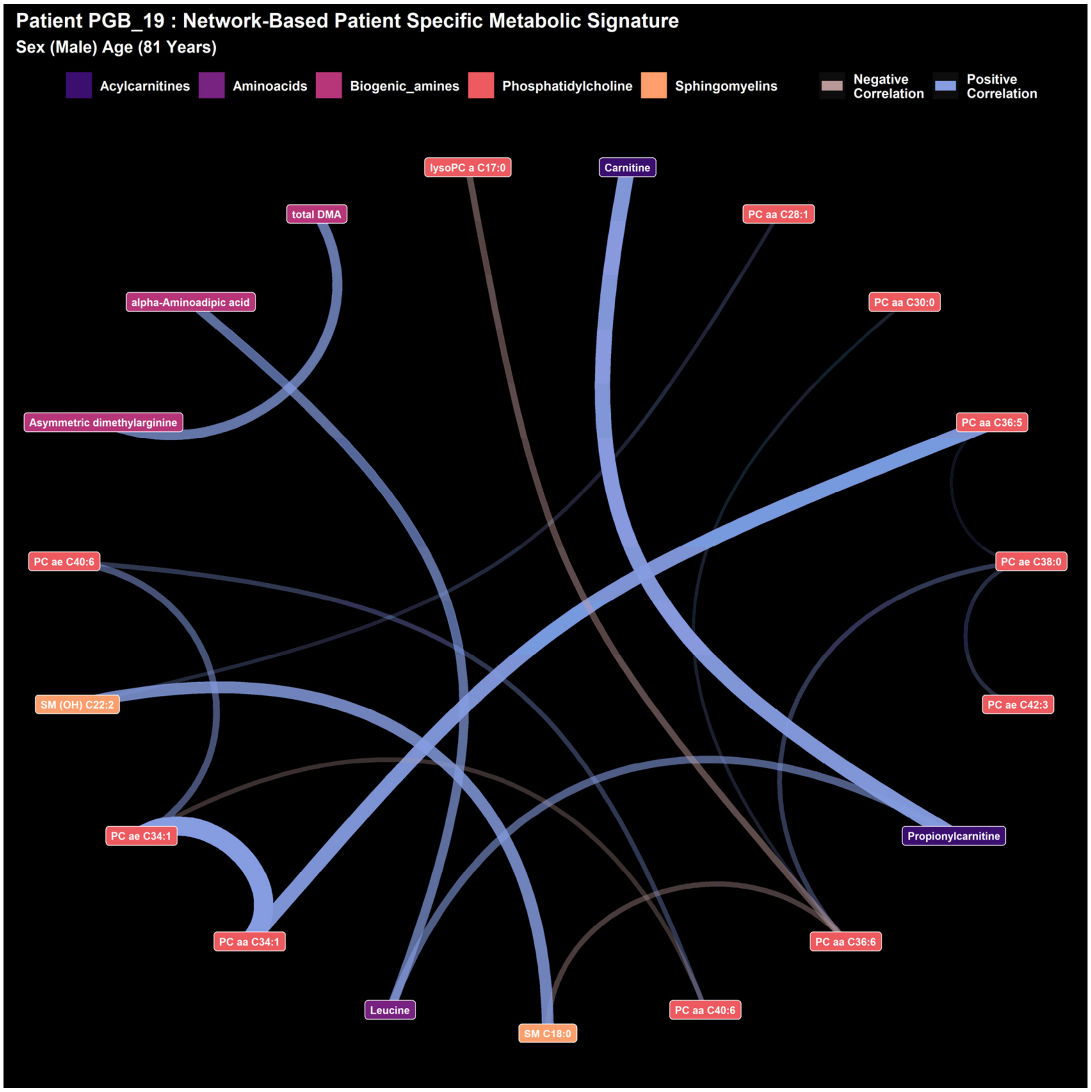

Figure S26. Network-based patient specific plasma metabolic signature (Patient PGB_19). The signature includes 19 metabolites; 11 phosphatidylcholines, 1 aminoacid, 2 acylcarnitines, 3 biogenic amines and 2 sphingomyelins.

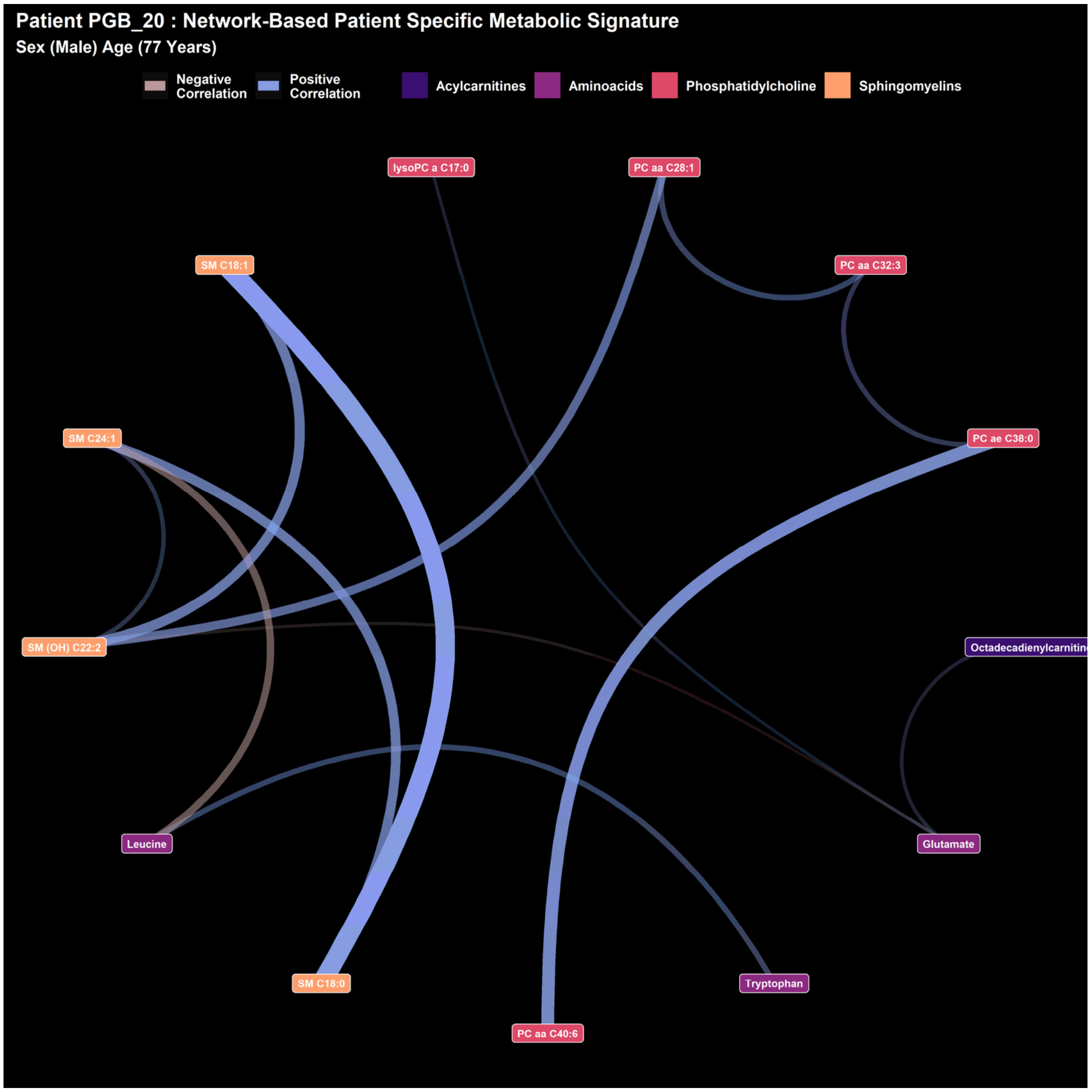

Figure S27. Network-based patient specific plasma metabolic signature (Patient PGB_20). The signature includes 13 metabolites; 5 phosphatidylcholines, 3 aminoacids, 1 acylcarnitine, and 4 sphingomyelins.

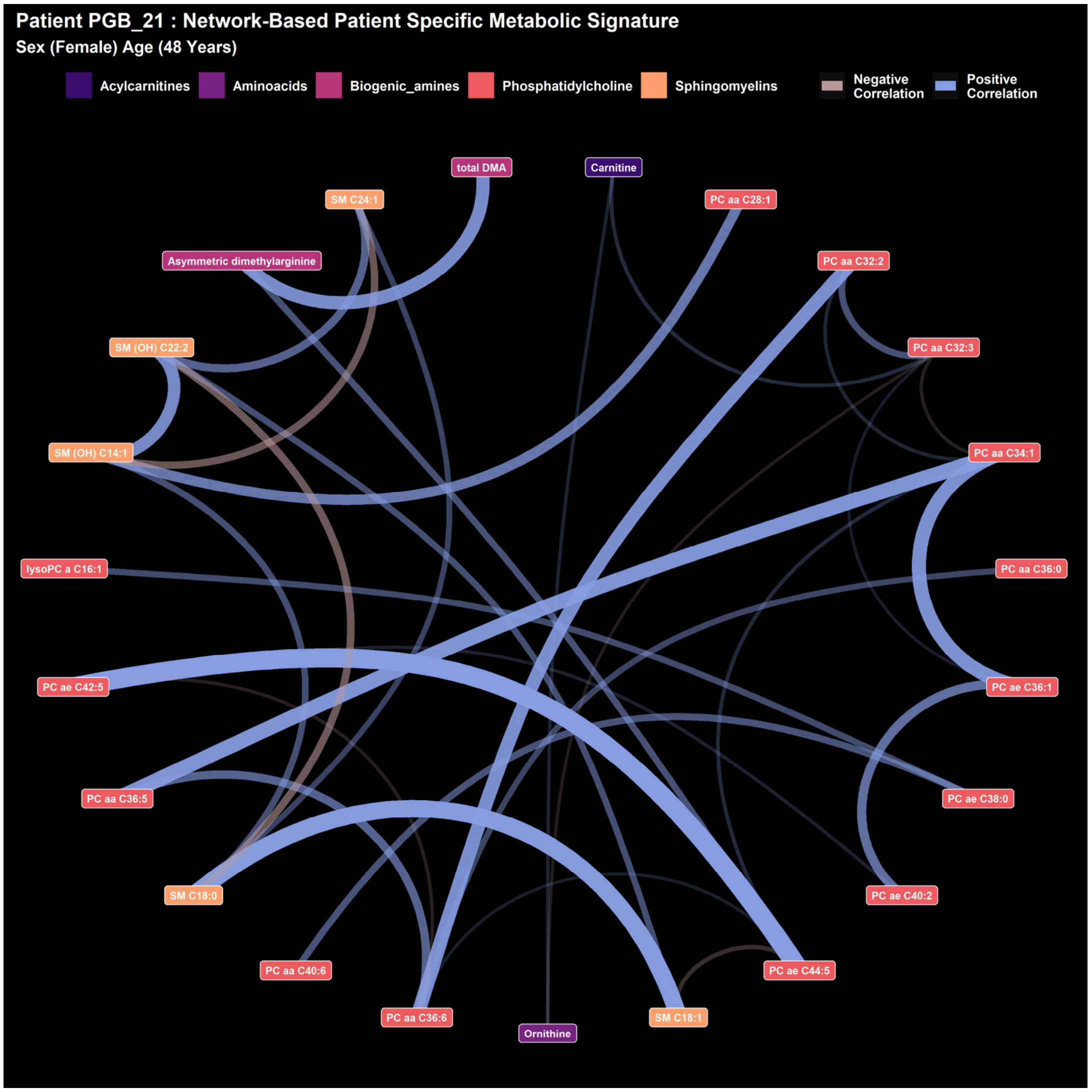

Figure S28. Network-based patient specific plasma metabolic signature (Patient PGB_21). The signature includes 23 metabolites; 14 phosphatidylcholines, 1 aminoacid, 1 acylcarnitine, 2 biogenic amines and 5 sphingomyelins.

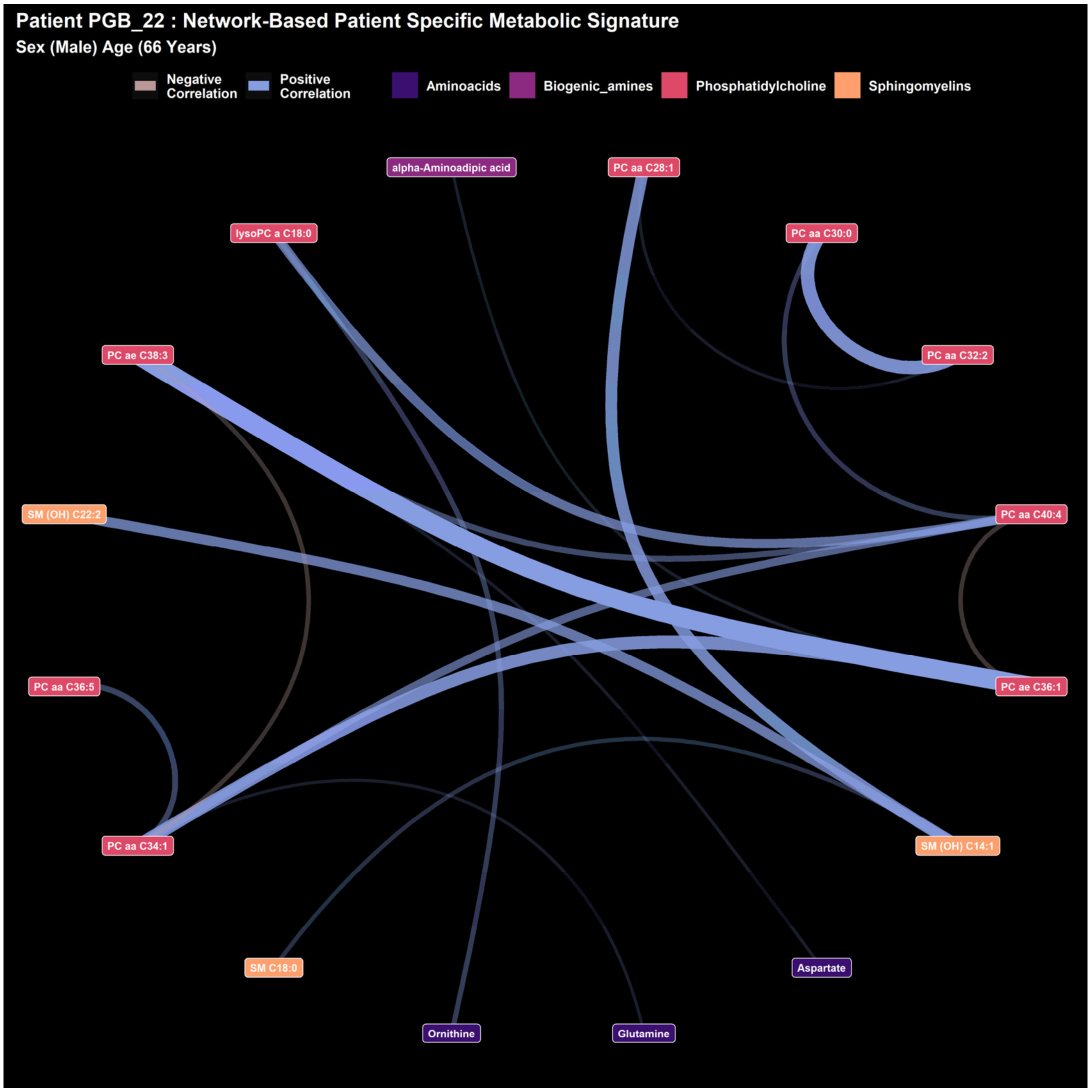

Figure S29. Network-based patient specific plasma metabolic signature (Patient PGB_22). The signature includes 22 metabolites; 9 phosphatidylcholines, 3 aminoacids, 1 biogenic amine and 3 sphingomyelins.

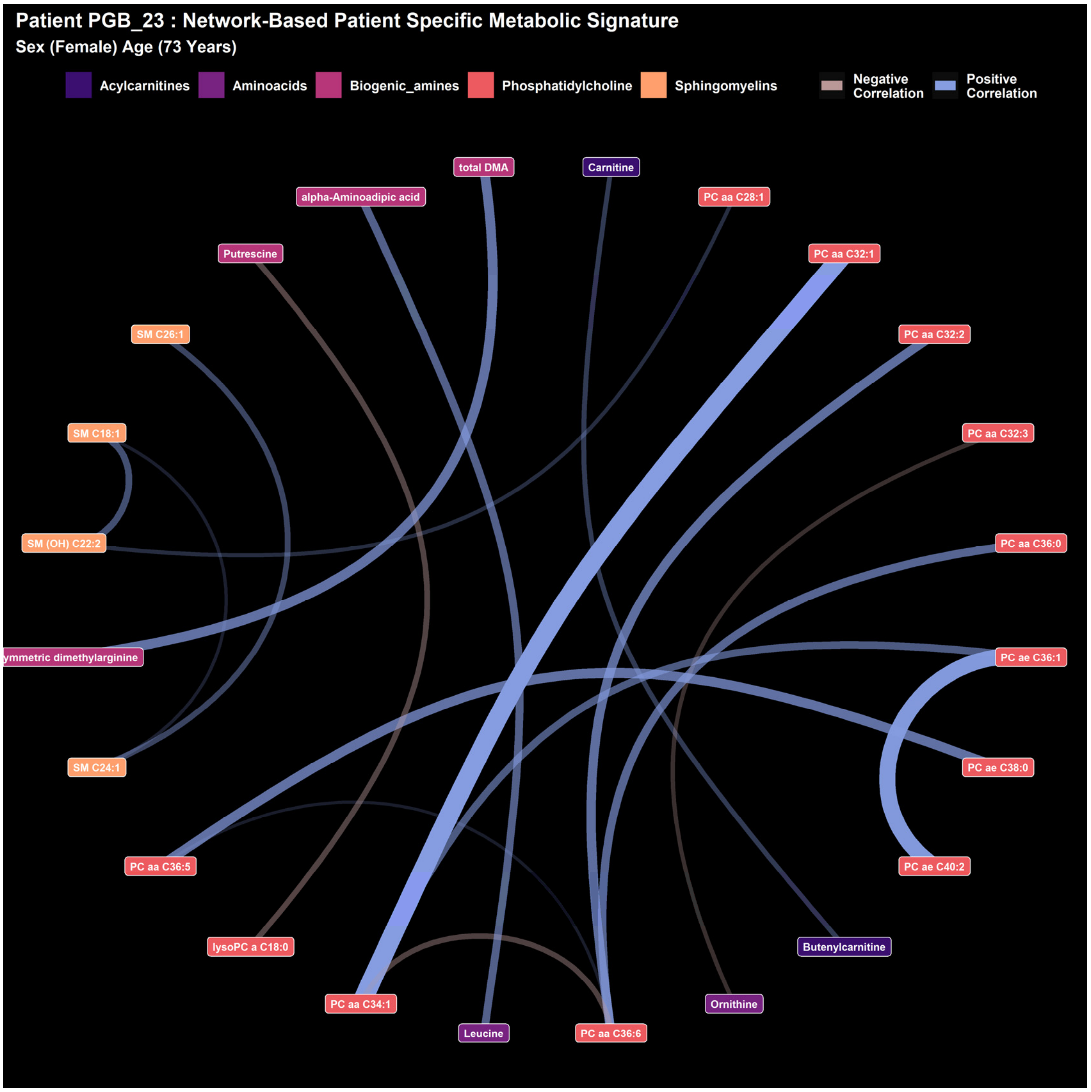

Figure S30. Network-based patient specific plasma metabolic signature (Patient PGB_23). The signature includes 24 metabolites; 12 phosphatidylcholines, 2 aminoacids, 2 acylcarnitines, 4 biogenic amines and 4 sphingomyelins.

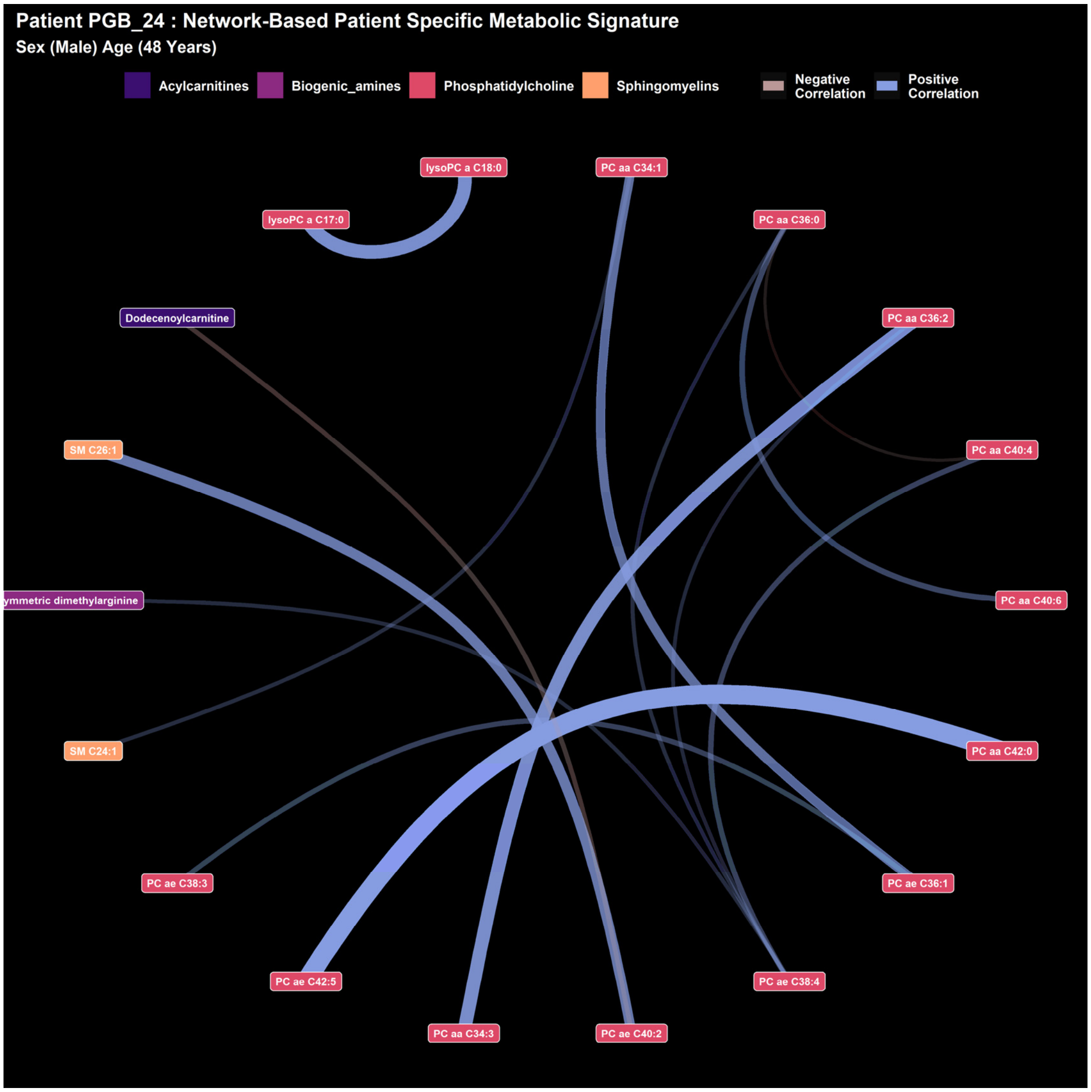

Figure S31. Network-based patient specific plasma metabolic signature (Patient PGB_24). The signature includes 18 metabolites; 14 phosphatidylcholines, 1 acylcarnitine, 1 biogenic amine and 2 sphingomyelins.

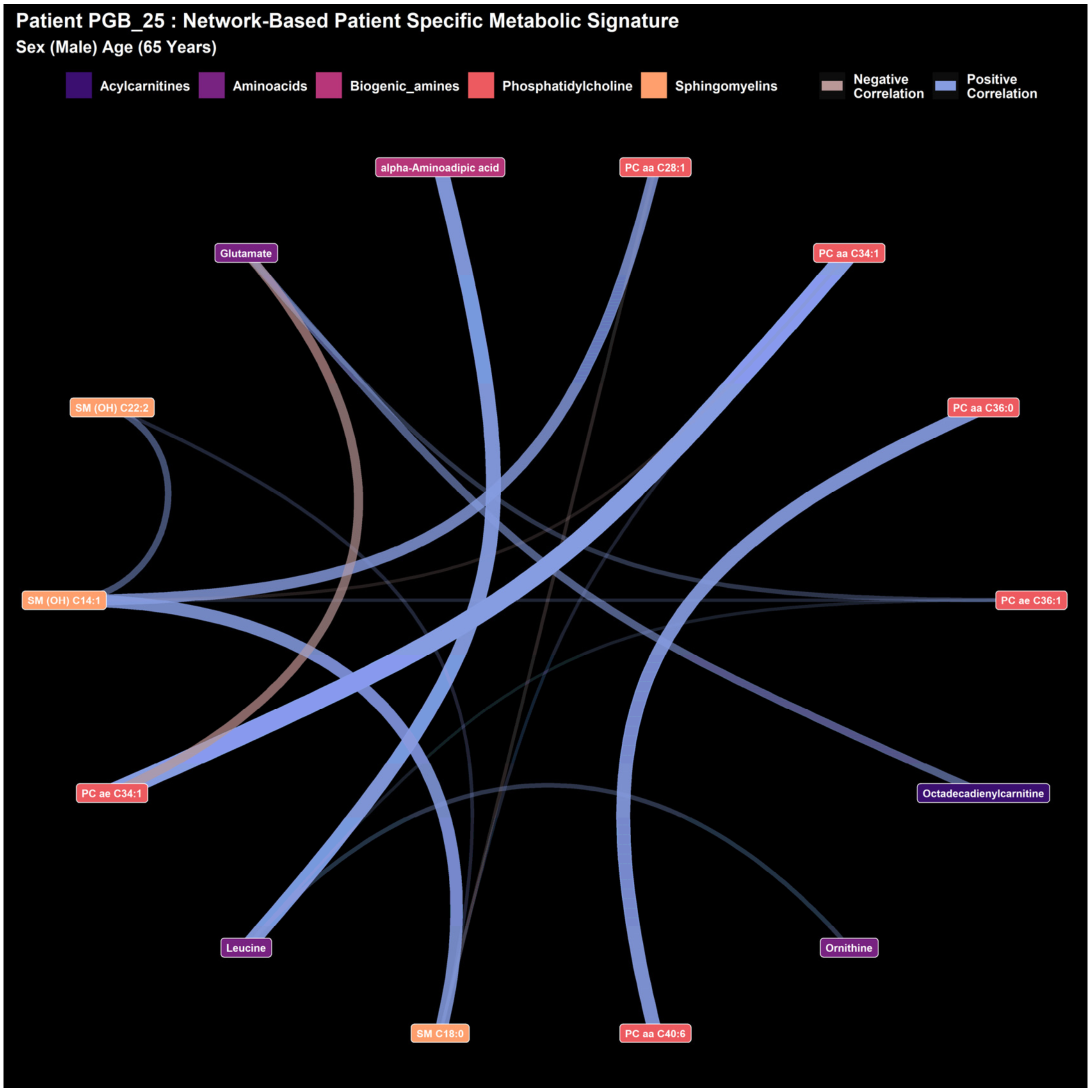

Figure S32. Network-based patient specific plasma metabolic signature (Patient PGB_25). The signature includes 14 metabolites; 6 phosphatidylcholines, 3 aminoacids, 1 acylcarnitine, 1 biogenic amine and 3 sphingomyelins.

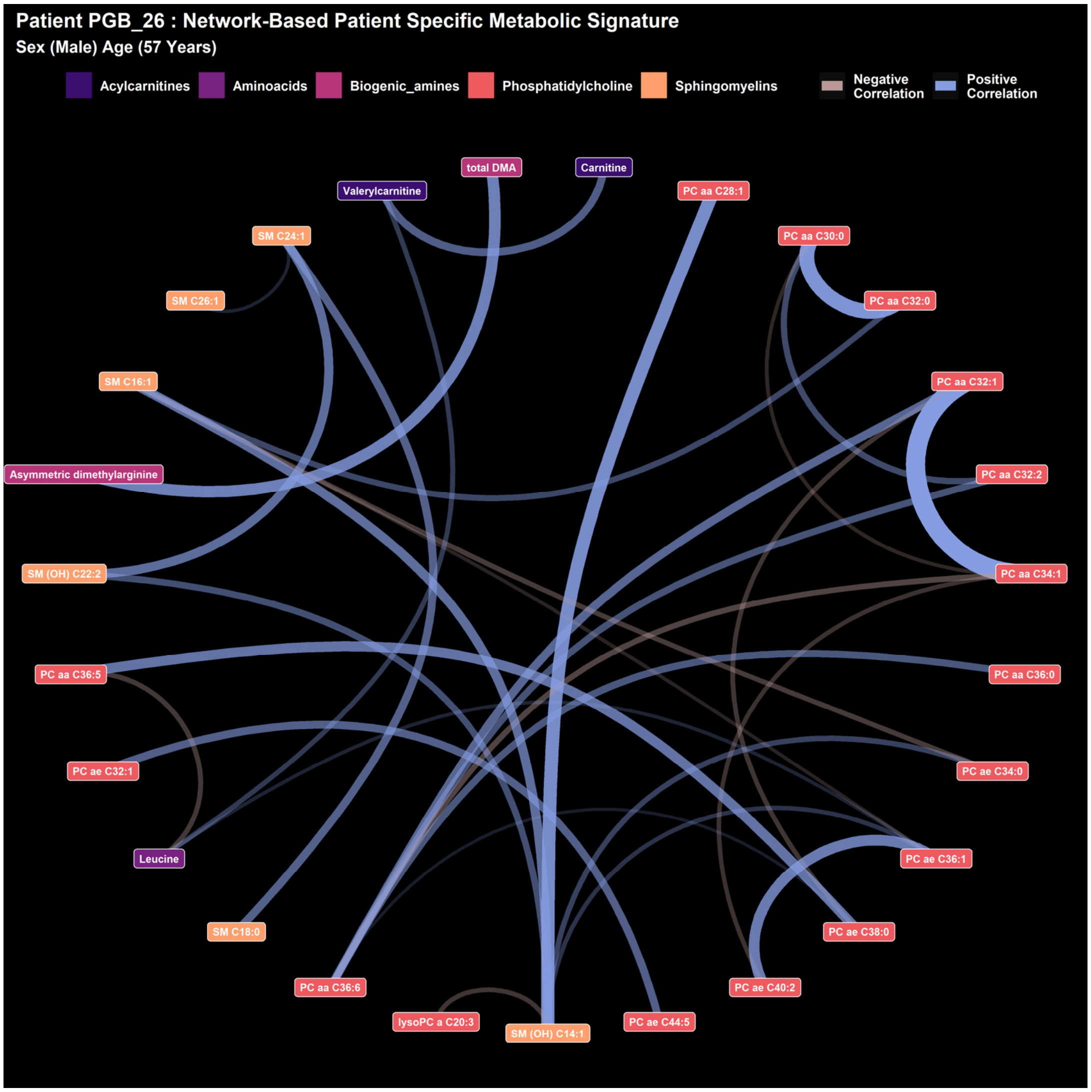

Figure S33. Network-based patient specific plasma metabolic signature (Patient PGB_26). The signature includes 27 metabolites; 16 phosphatidylcholine, 1 aminoacid, 2 acylcarnitines, 2 biogenic amines and 6 sphingomyelins.

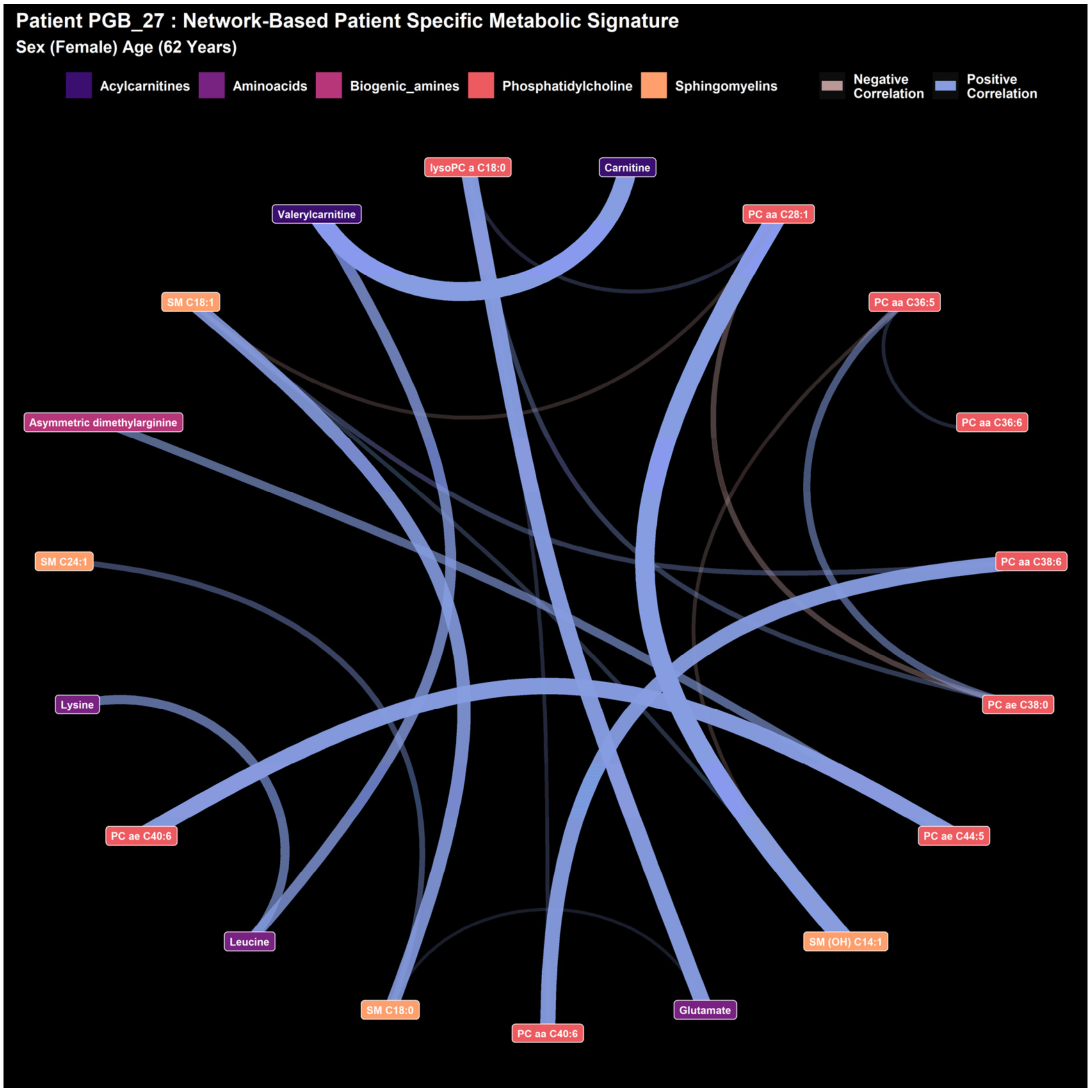

Figure S34. Network-based patient specific plasma metabolic signature (Patient PGB_27). The signature includes 19 metabolites; 9 phosphatidylcholines, 3 aminoacides, 3 acylcarnitine, 1 biogenic amine and 3 sphingomyelins.

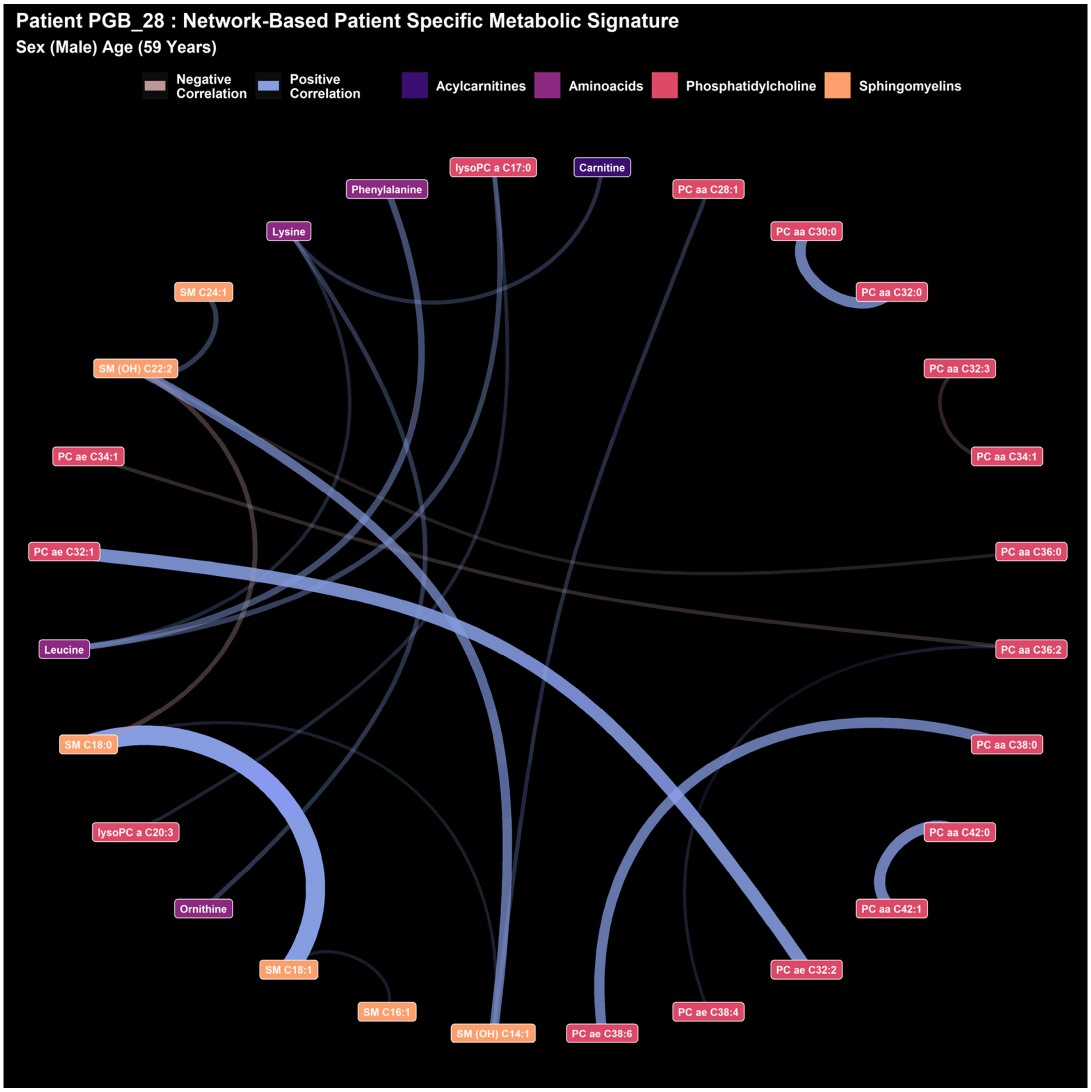

Figure S35. Network-based patient specific plasma metabolic signature (Patient PGB_28). The signature includes 28 metabolites; 17 phosphatidylcholines, 4 aminoacides, 1 acylcarnitine and 6 sphingomyelins.

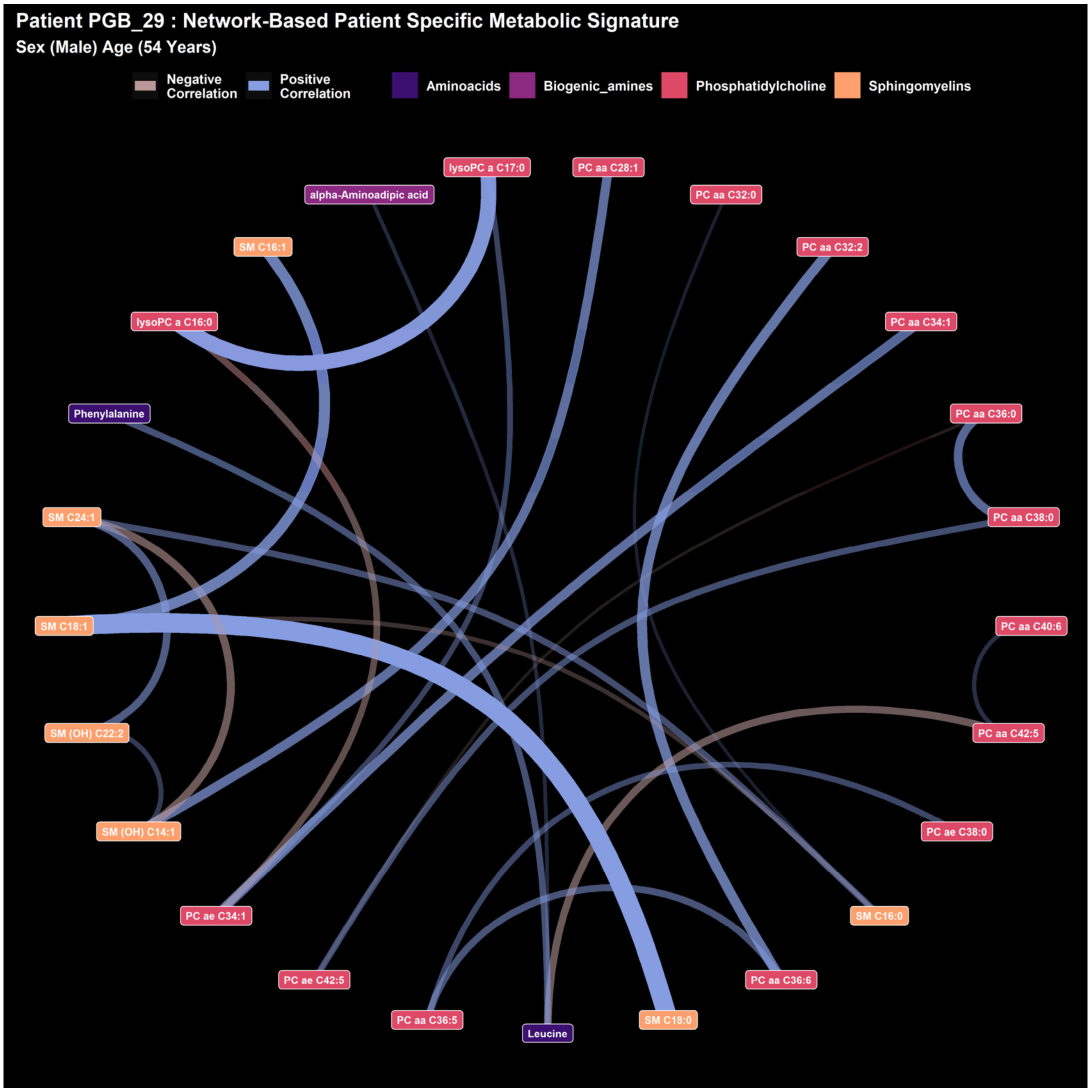

Figure S36. Network-based patient specific plasma metabolic signature (Patient PGB_29). The signature includes 25 metabolites; 15 phosphatidylcholines, 2 aminoacides, 1 biogenic amine and 7 sphingomyelins.
